# Retrenchment under climate-driven risks in subsistence farming communities

**DOI:** 10.1007/s11111-025-00493-8

**Published:** 2025-05-20

**Authors:** Nicolas Choquette-Levy, Dirgha Ghimire, Michael Oppenheimer, Rajendra Ghimire, Dil Ck

**Affiliations:** 1 Department of Geosciences, The Pennsylvania State University, University Park, PA USA; 2 Earth and Environmental Systems Institute (EESI), The Pennsylvania State University, University Park, PA USA; 3 Institute of Energy and the Environment (IEE), The Pennsylvania State University, University Park, PA USA; 4 School of Public and International Affairs, Princeton University, Princeton, NJ USA; 5 Institute for Social Research, University of Michigan, Ann Arbor, MI USA; 6 Institute for Social and Environmental Research–Nepal, Fulbari, Chitwan Nepal; 7 High Meadows Environmental Institute, Princeton University, Princeton, NJ USA; 8 Department of Geosciences, Princeton University, Princeton, NJ USA

**Keywords:** Climate risk, Risk perceptions, Smallholder farmers, Climate adaptation, Livelihood diversification, Nepal, Poverty traps

## Abstract

Increasing climate risks introduce new sources of uncertainty to smallholder farmers’ livelihood decisions. While farmers in different development contexts tend to accurately perceive long-term climatic trends, livelihood diversification as a climate resilience strategy has generally lagged behind awareness of climate risks. In this study, we investigate potential mechanisms behind this lagged response through a survey of 500 farming households in Nepal’s Chitwan Valley, a region that is highly dependent on subsistence agriculture and highly exposed to several climate-driven hazards. Specifically, we employ a suite of cross-sectional and time series econometric techniques to analyze how farmers’ information sources, social capital, and previous exposure to climate hazards shape climate risk perceptions and livelihood decisions. We find that climate-driven risks are highly salient to household perceptions of farming risks; however, they also drive higher perceived risks of common livelihood diversification strategies, including rural–urban migration and off-farm employment. Further, while farming households generally maintain diversified income portfolios, exposure to droughts and/or floods leads to persistent increases in the reliance on farming income, which we term a “retrenchment” response. We find evidence for both financial and psychological mechanisms behind this response, which may exacerbate environmentally driven poverty traps. Our results indicate that efforts to build farmers’ resilience to climate risks should especially account for perceived risks of livelihood alternatives, financial constraints, and loss-averse behavior in response to income shocks.

**Supplementary Information:**

The online version contains supplementary material available at 10.1007/s11111-025-00493-8.

## Introduction

Increasing climate risks over the coming decades are likely to threaten the livelihoods of many of the world’s 500 million smallholder farming households (i.e., those who farm on less than 2 ha of land) (Lowder et al., 2016; Morton, 2007; Howden et al., 2007). Such risks include the amount and timing of precipitation with respect to typical cropping cycles, the severity and frequency of droughts and floods, accelerating snowcap melting, and changes to mean and extreme temperatures (Intergovernmental Panel on Climate Change, 2022a, 2012). How farmers perceive and contend with such changes carries implications for several global sustainable development goals, including improving food security, preserving biodiversity, and managing rural–urban migration.

Here, we define climate risks as weather-related events that impact the economic success of farming activities, and whose frequency and/or severity will likely shift over the long term due to global climate change. While subsistence farmers have contended with high income volatility and natural hazards for decades (Dercon, 2002; Ellis, 1998), climate risks introduce new sources of uncertainty to livelihood decisions. For example, farmers face uncertainty regarding how swiftly natural hazard risks evolve (Arbuckle et al., 2015; Dang et al., 2012), which may affect decisions regarding whether to invest in coping strategies to manage short-term shocks or longer-term adaptive strategies (Singh et al., 2016). A second source of uncertainty is how climate-driven risks may affect farmers’ financial and natural capital. Over short time horizons, climate risks may actually increase farmers’ capital (e.g., through longer growing seasons or a particularly wet season that increases yields of staple crops) (Manandhar et al., 2011). However, over the longer term, climate impacts are likely to erode farmers’ assets through extreme events and unstable growing conditions, further constraining the capacity to respond to livelihood shocks. As most smallholder farmers rely primarily on agriculture for subsistence, climate shocks may further entrench poverty traps (Dasgupta, 1998; Bryan et al., 2014; Barrett & Carter, 2013) and lead to maladaptive strategies that can cascade to societal-wide shocks (e.g., involuntary displacement, deforestation, and food insecurity).

In response, governments at multiple scales have sought to build smallholder farmer resilience through strategies such as enhancing access to climate information, promoting climate-smart agricultural practices, and providing incentives for livelihood diversification through the development of small businesses and/or facilitating rural–urban migration (Aryal et al., 2020; World Meteorological Organization, 2014; Nepal Ministry of Agricultural Development, 2015). Yet, research conducted in different parts of the world demonstrates mixed evidence regarding the effectiveness of such efforts. While farming households in East Asia, South Asia, and Southern Africa have exhibited varying degrees of income diversification through migration and planting different crops, this effect is mainly observed in response to long-term, repeated exposure to natural hazards (Ma & Maystadt, 2017; Antonelli et al., 2022; Arslan et al., 2021). By contrast, such households exhibit little diversification, and sometimes increased specialization, in the immediate years following exposure to natural hazards (Ma & Maystadt, 2017; Antonelli et al., 2022; Wuepper et al., 2018). Further, while farmers generally accurately perceive long-term climatic trends (Manandhar et al., 2011; Truelove et al., 2015; Bro, 2020; Thapa & Dhakal, 2024), limited access to credit, land, and information often prevents them from proactively deploying adaptive strategies, even when they perceive high climate risks (Bro, 2020; Mulwa et al., 2017; Tessema et al., 2018). In some cases, farmers have also discounted negative climate forecasts (Grothmann & Patt, 2005; Ziervogel, 2004) or trusted that public adaptation measures would be sufficient to manage climate risks (Dang et al., 2014).

One under-explored mechanism for a slow diversification response could be that climate hazards influence risk perceptions not only of farming strategies, but also livelihood alternatives such as migration and off-farm employment. This is particularly true for loss-averse farmers that are closer to the survival threshold and who do not have psychological appetite for experimentation (Bryan et al., 2014; Tessema et al., 2018; Sagemüller & Musshoff, 2020). This study therefore seeks to elaborate mechanisms by which climate-driven risks shape farmer perceptions of various livelihood strategies and the extent to which these are related to income diversification strategies. Specifically, we seek to investigate two main research questions: (i) What factors lead to (the lack of) livelihood diversification among smallholder farmers in response to climate risks? (ii) How are farmer perceptions of climate risks related to their perceptions of livelihood diversification options?

To investigate these questions, we administered a cross-sectional, face-to-face survey of 500 farming households in Nepal’s Chitwan District from May to July 2022. The agricultural sector in Nepal represents an important case study to better understand how smallholder farming communities may adapt to climate risks. While the country is undergoing a rapid urbanization process, as of 2021 agriculture still accounts for 21.3% of Nepal’s GDP and 64% of its employment, far higher than regional (16.7% and 42%, respectively) and global averages (4.3% and 27%, respectively) (World Bank Group, 2021). Nepal faces several substantial climate risks, including altered monsoonal patterns that affect the timing and volume of precipitation (Aryal et al., 2020), warming temperatures that outpace global trends and affect soil fertility (Karki & Gurung, 2012; Luitel et al., 2020), and increased potential for catastrophic events that can wipe out harvests and homes (e.g., glacial lake outburst floods) (Nepal Ministry of Forests and Environment, 2019; Intergovernmental Panel on Climate Change, 2022b). Most of the country’s farmers operate small-scale farms (average size of 0.7 hectares) that rely on rainfed agriculture (Nepal Ministry of Agriculture, Land Management, and Cooperatives, 2018), limiting the resources and capital that they can deploy to adjust to changing environmental conditions. Gaining a better understanding of the factors that influence Nepali farmer risk perceptions and livelihood decisions can provide useful insights for other Global South agricultural contexts that may face similar threats in the coming decades.

## Methods

### Study area and survey design

The geographic focus of our study, the Chitwan District, is one of Nepal’s main agricultural regions, cultivating a variety of subsistence and cash crops, including rice, wheat, maize, and several fruits and vegetables. Most households also supplement crop harvests with livestock ranging from capital-intensive buffalo and cows to less-expensive goats and chickens. In Nepal’s 2010 National Adaptation Plan of Action, the Chitwan District ranked “High” (fourth out of five categories) on an index of overall vulnerability to climate change, reflecting high exposure to increasing droughts, floods, and pests, among other hazards (Nepal Ministry of Environment, 2010). From 1970 to 2019, the average annual temperature in this zone has increased by 0.22 $^{\circ }$C per decade, while average annual precipitation has declined by 19.8 mm per decade (Luitel et al., 2020). These rates of climatic change are roughly double in magnitude compared to global mean trends in temperature (+0.1 $^{\circ }$C per decade) and precipitation ($+5.4$ to 11.1 mm per decade) over similar periods (Intergovernmental Panel on Climate Change, 2021). Previous surveys have indicated that a majority of Chitwan farmers accurately perceive the direction of these long-term trends and also perceive more intense rainfall during monsoon periods (Thapa & Dhakal, 2024). Demographically, the region is home to a diverse mix of ethnic and caste groups, including Brahmin, Chetri, Dalit, Gurung, and Indigenous Tharu and Janjati populations. Additionally, over the last 20 years, the Chitwan District has seen a marked increase in outmigration to several countries, including India, Saudi Arabia, Qatar, and East Asian countries (Massey et al., 2010; Ghimire et al., 2019, 2021). Geographically, the 15 km by 30 km region is located in the Terai plains and is transected by two main rivers, the Narayani and East Rapti, with different propensities to flood during the monsoonal rains. This study site therefore provides a high degree of heterogeneity in livelihood strategies, exposure to climate risks, ethnic/caste identity, and connection to migrant and other social networks that allow us to investigate a variety of factors hypothesized to underlie risk perceptions and climate adaptation strategies.

**Fig. 1:**
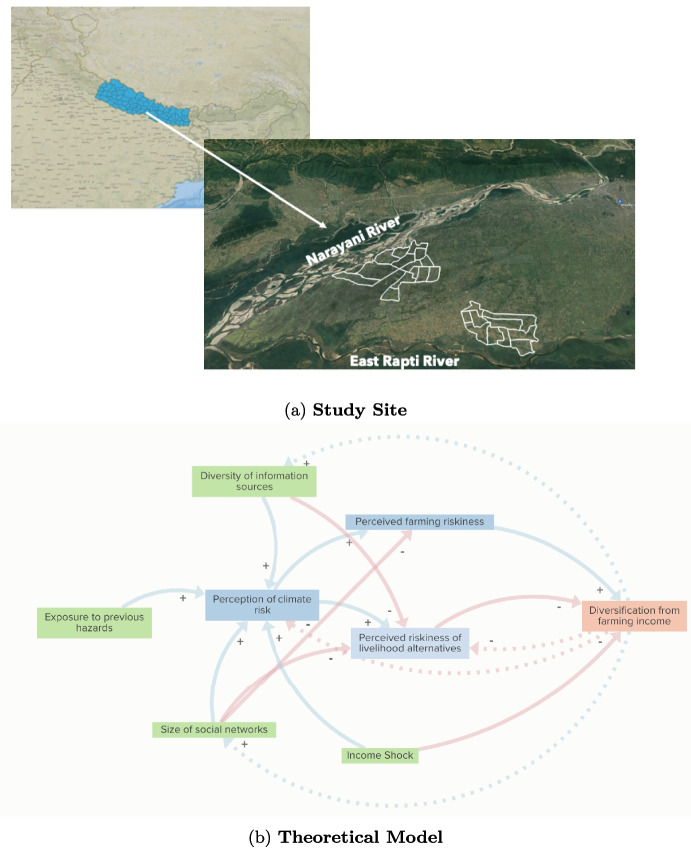
Study site and conceptual framework for analytical strategy. **a** Map illustrates location of Chitwan District in south-central Nepal, and of the wards within Chitwan District sampled in this survey (white boundaries). **b** Arrows indicate hypothesized relationships between independent variables (green), intervening variables (blue), and dependent variables (red). Where applicable, a $+$ or − sign indicates the hypothesized directional effect of a relationship. Dashed lines represent potential feedback relationships

Participants for our survey were recruited from two rural wards of Chitwan’s main metropolitan city, Bharatpur, with one bordering the larger, more flood-prone Narayani and the other bordering the smaller, less flood-prone East Rapti; the geographic centers of the wards are 7 km apart (Fig. 1a). In each ward, we stratified the sample by randomly selecting 200 households within 1 km of the riverbank and 50 households at least 3 km from the river.^1^ In Nepal, districts and municipalities are responsible for passing local laws, which are then implemented by wards; we can therefore assume consistent agricultural and development policy conditions across all wards in Bharatpur municipality. This sampling strategy allows us to exploit localized variation in exposure to climate hazards while controlling for similar economic and political conditions, strengthening the inferential power of our research design with respect to the effects of hazard exposure, social capital, and climate risk perceptions on livelihood diversification strategies. However, our inferences are limited to observing household-level variation in our variables. Absent a controlled or natural experiment, our results may still be subject to omitted variable bias (e.g., differences in respondents’ ability to accurately recall information), and we do not claim to demonstrate causality.

**Table 1:** Demographic summary statistics

Variable	2022 Survey Population	2021 Census Chitwan District	2021 Census Nepal Population
Total individuals	2389	719,859	29,164,578
Households	500	179,345	6,666,937
Average household size	4.78	4.01	4.37
Gender			
Female	62.8	51.1	51.1
Male	37.2	48.9	48.9
Age (Pct of adult population)	45.0 (median)		
18–34	22.0	44.6	42.5
35–44	30.6	19.6	20.8
45–54	22.2	14.8	15.7
55–64	16.4	10.5	10.7
65+	7.4	10.5	10.4
Annual Income (NRs)	29,800 (average)		
0–100,000	17.4		
100,000–250,000	32.2		
250,000–500,000	31.8	N/A	N/A
500,000–1,000,000	15.4		
1,000,000+	3.2		
Educational Attainment (Grade)	5.48 (avg grade)		
0–5	48.2	28.8	33.1
6–10	43.8	33.0	35.4
SLC-Intermediate	6.2	27.8	22.5
Bachelor’s or above	1.8	7.8	6.7
Caste			
Brahmin-Chetri	35.8	39.8	28.5
Newar	1.8	4.9	4.6
Gurung-Magar-Tamang	12.4	10.6	14.4
Dalit	15.0	N/A	N/A
Tharu-Darai-Kumal	31.4	6.7	6.7
Other	3.6	38.1	45.8

To assess representativeness of our survey sample, we compare key demographic variables for the survey sample with 2021 Nepali Census data for the Chitwan District and for Nepal nationally (Nepal National Statistics Office, 2023). There are several important demographic differences between the survey sample and both populations (Table 1). First, there were markedly more female respondents in the survey (62.8%) compared to the sex composition of the Census (51.1%). Survey respondents also skewed older, with only 22% of respondents between ages 18 and 34 (whereas this age group comprises 45% of Chitwan adults). Respondents had less formal educational attainment than either Chitwan or the overall Nepali population, with roughly half the survey sample not having received secondary education. With respect to caste identity, Dalit and Tharu/Darai/Kumal respondents were more highly represented in the sample than the broader Nepali population. These castes have historically had lower socio-economic status compared to other caste identities.

Differences between our survey sample and broader population data likely reflect three key sampling criteria: (i) restricting our survey sample to farmers, and excluding Chitwan residents in other occupations who do not have a farm and are likely to skew younger and more highly-educated; (ii) restricting our sample to farmers who are currently residing in Chitwan during the period of data collection (May–July 2022), which excluded migrants from the region living elsewhere and who are likely to skew younger and more male; and (iii) sampling wards that were likely to have experienced climate-related hazards in recent years, in which residents may skew poorer and from less advantaged castes/ethnic identities than residents of less-exposed wards. While we cannot claim large-scale representativeness for the entire Nepali population, our sample consists of demographic groups—smallholder farmers with generally lower than average socio-economic status—that are most likely to be characterized by high exposure and vulnerability to climate change. More broadly, our survey sample shares important characteristics with rural communities across much of South Asia and sub-Saharan Africa, including a reliance on subsistence agriculture, aging population due to high rates of youth out-migration, high rates of extreme poverty, and high exposure to climate risks (Aryal et al., 2020). At the same time, smallholder farming communities in Nepal differ from others in South Asia in part on their low access to credit and banking systems, and high reliance on international, rather than local, migration (Aryal et al., 2020; Solomon et al., 2024).

Survey questions were designed to measure several categories of independent, intervening, and dependent variables, and were refined after pre-testing questions through 12 semi-structured interviews with farmers across the Western Chitwan Valley. While many variables were assessed through a cross-sectional design, we also asked respondents to recall their exposure to various natural hazards (including droughts, floods, excess heat, pests), livelihood activities they engaged in (including farming cereal crops, farming fruit/vegetable crops, raising livestock, engaging in non-farm jobs, engaging in rural–urban migration), and income earned from these activities for each year from 2015 to 2021. These variables were assessed using a life history calendar method, in which respondents are presented with a physical calendar that contains Nepali years and memory cues of locally relevant events. This design was developed in previous demographic surveys of the Chitwan Valley Family Study, a 28-year panel study of this area, which have demonstrated its efficacy in improving respondent recall of various life history events, including migration trips, farming choices, marital events, and experience with mental disorders (Axinn et al., 1999, 2020; Brauner-Otto et al., 2020; Ghimire et al., 2021; Brauner-Otto et al., 2022). In particular, this version of the life history calendar contained significant natural hazard events (the 2015 Nepali earthquake), political events (a local election in 2017), and societal events (the onset of COVID-19 in 2020) as cues to help respondents situate their personal life history and household events in an accurate chronological order.

### Theoretical framework

In addressing our research questions, we form hypotheses based on three theoretical frameworks that are especially relevant to how subsistence farming households perceive and act on climate risk: Protection Motivation Theory (PMT), the New Economics of Labor Migration (NELM), and Security-Potential/Aspiration (SP/A). Each theory focuses on a different timescale and scope of decision-making; we combine them to develop hypotheses about climate risk and livelihood diversification. First, PMT applies broadly to a range of contexts in which decision-makers perceive a threat (including health, safety, and economic threats), and states that individuals act to mitigate the risk of such threats as a function of the perceived severity of a threat and the perceived capacity to mitigate this risk (Rogers & Prentice-Dunn, 1997; Dang et al., 2012; Arbuckle et al., 2015). Therefore, we should expect that the more farming households perceive climate change as a threat, and/or the more that households believe they have sufficient resources to mitigate climate hazards, the more likely they should be to take observable forms of climate adaptation, including livelihood diversification (Grothmann & Patt, 2005). This leads to our first hypothesis, which applies to farming households’ long-term income diversification strategies:

- H1: *Farmers that generally perceive higher climate risks are more likely to exhibit diversified household income portfolios and therefore rely less on farming for their income.*

A second relevant theory comes from the New Economics of Labor Migration (NELM), which applies more specifically to long-term livelihood decisions in rural development contexts. It postulates that households in such contexts seek to minimize risks to their livelihoods and overcome constraints in access to financial capital (Stark & Bloom, 1985). A common strategy to cope with risk is to diversify sources of household income by engaging in multiple livelihood strategies, including rural–urban migration (Lucas & Stark, 1985). This principle supports H1: if households perceive elevated risks to farming due to climate change, especially in emerging market contexts with limited access to financial services, then they should be more likely to diversify their livelihood portfolios. Additionally, households aim to minimize their sense of relative deprivation (i.e., a perceived gap between their well-being and that of others in their social network) (Massey et al., 1993). Conversely, households with smaller social networks may be less motivated to change livelihood strategies. This leads to a second hypothesis:

- H2: *Farmers with greater access to social and informational capital are more likely to diversify income sources away from farming in response to climate-related shocks.*

While NELM and PMT provide testable implications for whether farming households take observable actions to reduce climate risk, they do not differentiate between the different risk management options that farmers may choose from. By contrast, Security-Potential/Aspiration (SP/A) (Lopes & Olden, 1999) provides more granular theory on how decision-makers choose among a portfolio of risky options. A significant feature of SP/A is that decisions are framed as a dual-objective process. First, individuals assess their potential gains or losses relative to an aspiration. If the outcomes of a decision are unlikely to meet a basic aspiration level, a security-minded individual may emphasize a familiar strategy in order to minimize or eliminate a loss. This mechanism echoes loss-averse behavior in Cumulative Prospect Theory, in which decision-makers tend to disproportionately penalize the options that may lead to utility losses compared to utility gains of equivalent magnitude (Tversky & Kahneman, 1991). However, if the aspiration level has a good chance of being met, the individual then reverts to traditional risk-averse behavior, in which decision-makers generally prefer options with more certain outcomes to those that are perceived as more volatile.

There is some empirical evidence for this theory: coffee farmers in Nicaragua were found to exhibit higher levels of risk aversion when experiencing food insecurity, even when controlling for income (Bro, 2020), and smallholder farmers in Southeast Asia who had experienced more income shocks (including drought- and flood-induced) exhibited more pronounced loss-averse behavior (Sagemüller & Musshoff, 2020). This framework may have particular relevance for farmers evaluating multiple adaptation options in light of climate shocks that threaten their basic subsistence, especially if those adaptation strategies are themselves perceived as risky and uncertain compared to farming familiar staple crops (Umar, 2014; Lipion, 1968). Specifically, when exposed to a shock that threatens a food production aspiration (e.g., a drought or flood), smallholder farmers may be expected to double down on farming in order to minimize or eliminate their losses, whereas in the absence of climate shocks, farmers may be more willing to diversify risks through alternative livelihoods. Our last formal hypothesis is therefore:

- H3: *In years in which farming households experience a climate-related shock (e.g., a drought or flood), they will be less likely to diversify income sources away from farming.*

Informed by these principles, Fig. 1b summarizes our hypothesized relationships between independent (green), intervening (blue), and dependent (red) variables. Below, we employ a three-stage analytical design to test these hypotheses. First, we assess respondents’ perceptions of climate risks based on how they foresee such risks evolving in the next 5 years and the perceived salience of these factors to their economic success. Next, we estimate the degree to which farmer climate risk perceptions are salient to their general risk perceptions of different livelihood options, including farming cereal crops, investing in livestock, engaging in migration, and seeking off-farm employment. Finally, we estimate the effects of climate risk perceptions, exposure to droughts and floods, and access to information and social capital on households’ income sources via cross-sectional and time series models.

### Construction of key variables

In this section, we describe important methodological choices in operationalizing key variables in our analytical design and provide further information in SI 2.

#### Social and informational capital

To measure social capital, we asked respondents to indicate how many times per year someone from their household has participated in each of eight different types of social groups present in the Western Chitwan Valley (e.g., a women’s group, youth group, farming cooperative, and other options). As an alternative measure, we also ask respondents to indicate the number of friends with whom they discuss matters related to farming, migration, and other livelihood decisions. Finally, we measure informational capital by asking respondents to identify how often they consult each of 12 types of information sources, including radio, television, agricultural offices, social media, relatives and friends, and other options. For each form of capital, we construct an index that reflects how many sources a respondent consults/participates in over the course of a year.

#### Climate risk perceptions

**Fig. 2:**
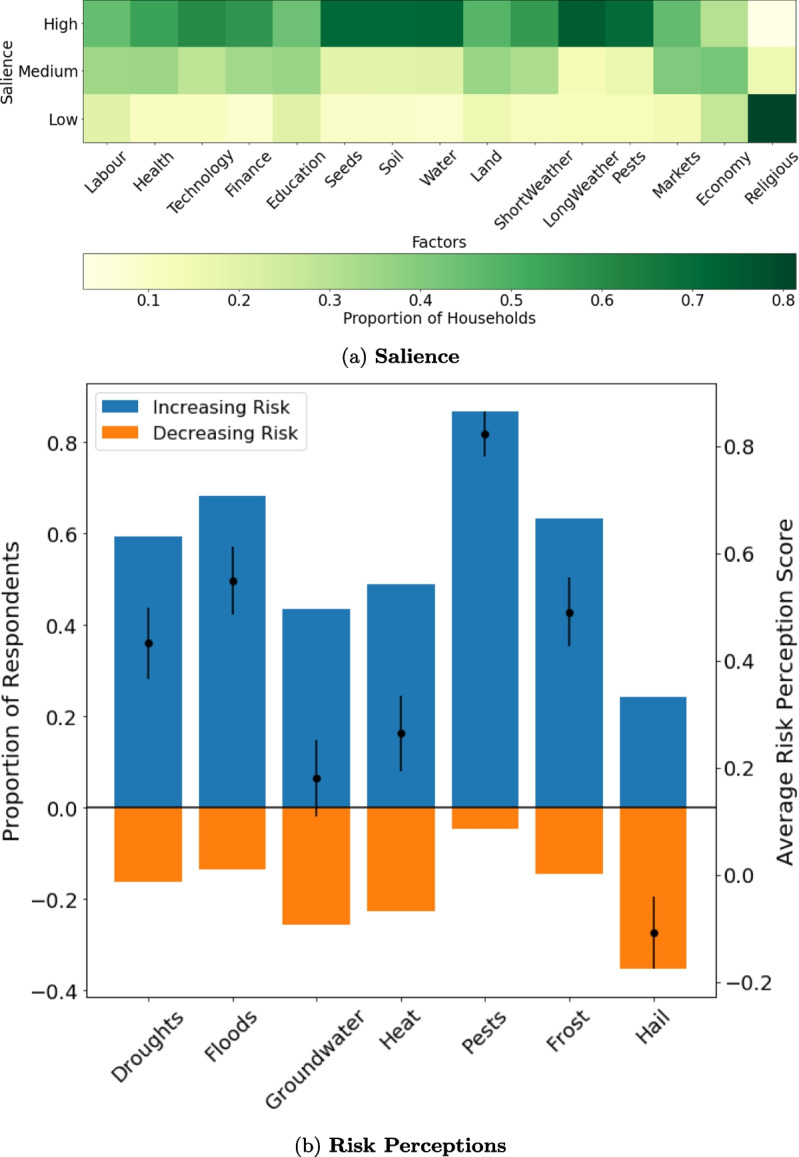
**Summary statistics on perceived climate risk salience and direction.** **a** The heat map illustrates the distribution of survey respondents by the level of salience assigned to each factor on the x-axis with respect to their economic success. For a given factor, the proportion of respondents assigning “Low,” “Medium,” or “High” importance to a given factor is illustrated by green shading. **b** Survey respondents generally find that most climate hazards are likely to worsen over the next 5 years. For each hazard on the x-axis, blue bars indicate the proportion of respondents projecting the hazard’s impacts to become worse over the next 5 years, and orange bars represent the proportion of respondents projecting a hazard’s impact to decrease in risk over the next 5 years. Proportions may not add to 1, as respondents could also answer that “no change” was likely in the hazard. Black dots indicate the sample-wide mean score for each hazard, with 1 representing 100% of respondents believing a hazard will diminish in impact, and $-$1 representing 100% of respondents believing hazards will get better. Error bars indicate the 95% confidence interval. Summary statistics are shown for $N=500$ observations, with the exception of the Droughts column in **b**, which summarizes $N=499$ observations. One respondent answered “Don’t Know” to this question

The main intervening variable in our research design is farmers’ perceptions of climate risk to their livelihoods. To operationalize this variable, we draw from existing climate risk perception indices (Dang et al., 2012; Waldman et al., 2019; Zander & Garnett, 2020) and measure two dimensions of perceived climate risk: (i) the degree to which respondents perceive that climate-driven hazards are likely to improve or worsen in impact over the near term and (ii) the salience of climatic factors to respondents’ economic and adaptation decision-making (Fig. 2). Similar to Waldman et al. 2019, we measure the first indicator by focusing on respondents’ perceived risk of future climate-driven hazards for their crop harvests through the question: *“Over the next five years, how do you think the impact of [X hazard] will change, compared to today?”* Following the approach of Dang et al. (2012) in aggregating perceptions along several dimensions of climate risk, we construct a directional risk perception index, $D_i$, by assigning a score of +1 to each hazard that household *i* identified as becoming more severe in the future, and $-$1 for each hazard the household identified as becoming less severe. We standardize this measure such that $\tilde{D}_i$ can take on values in the interval $[-1,1]$, with negative values indicating a general perception that climate risks are likely to alleviate in the near future and positive values indicating a general perception that risks are likely to worsen in the near future (SI 2.2). We note that $\tilde{D_i}$ represents an aggregate measure of the directionality of climate risk, and respondents may perceive certain hazards to be worsening while believing the impacts of other hazards are staying constant or alleviating. In SI 4.1, we conduct a disaggregated analysis of how perceptions of individual climate-linked hazards relate to perceptions of major livelihood strategies.

To measure the salience of climate-related risks, we follow Zander and Garnett (2020) in asking respondents to assess the relative salience of a variety of factors (e.g., health, access to labor, weather conditions) to their livelihood decision-making through the question: *“How significant would you say [X factor] has been in determining your level of economic success from growing crops?”* We then calculate the salience of climate-related risks ($\tilde{S}_i$) by comparing respondents’ assessment of “long-term weather conditions” (defined for respondents as conditions lasting more than two weeks) with how they assessed each of the other 14 factors. To avoid potential biases due to the availability heuristic (i.e., respondents may over-weigh factors that first come to mind), we asked respondents this set of questions before engaging in any climate- and weather-specific questions. We standardize respondents’ answers to these questions such that $\tilde{S}_i$ takes values between 0 (climate factors are not at all salient) and 1 (climate factors are highly salient).

Finally, we combine information on the salience of climate factors and the perception of future climate risks through a composite climate risk perception indicator, $\tilde{R}_i$:

1 $$\begin{aligned} \tilde{R}_i = \tilde{D}_i * \tilde{S}_i \end{aligned}$$

This composite index is different from previous indices reported in the climate risk literature, but captures several characteristics that facilitate analysis of our research questions. First, it takes on values in the interval [$-$1, 1], and the direction indicates whether the respondent believes climate risks will alleviate ($\tilde{R}_i < 0$) or worsen ($\tilde{R}_i > 0$) in the near future. Second, the magnitude is an indicator of how much of an effect a respondent believes future climatic conditions are likely to have on their farming success. A value $\tilde{R}_i \approx 1$ indicates that the respondent believes most climate-driven hazards will worsen in the coming years, and that this is highly salient to their farming success. However, if a respondent believes that only a few hazards may worsen, and/or that long-term weather conditions are not particularly salient to their farming success, then $\tilde{R}_i$ will take on lower values.

#### Observed livelihood diversification

To assess our main dependent variable, livelihood diversification, we used the calendar format to ask respondents whether they had deployed each of 14 types of livelihoods over the Nepali years 2072–2078 (roughly, 2015–2021), and if so, approximately how much income their household had earned from each livelihood for that year. This generated a quasi-panel dataset of livelihood choices and incomes for 500 farming households that respondents recalled over 7 years, which allows us to estimate the composition of household incomes as a function of exposure to hazards and the independent and intervening variables described above. For the purposes of analysis, we aggregated the 14 livelihoods into five general categories: farming, raising livestock, engaging in off-farm jobs within the Chitwan District, migration, and other (e.g., government pensions, see SI 2.3 for full list of livelihoods and their generalized categories). We measure livelihood diversification by evaluating the proportion of income derived from farming crops (including cereals, fruits, and legumes) vs. other livelihoods. In additional analyses, we also examine how the proportions of income derived from common farming alternatives (e.g., off-farm employment and migration remittances) are affected by exposure to and perception of climate risks.

### Econometric specifications

We test our hypotheses through three main econometric analyses. First, we measure the salience of perceived climate risks to generalized risk perceptions of four livelihood strategies: farming cereal crops, raising cattle and buffalo, working off-farm wage labor jobs, and migrating internationally. These are the four livelihood options that respondents found riskiest on average (SI 3.2). In this analysis, the dependent variable is respondents’ perception of livelihood risk, expressed on a Likert Scale ranging from 1 (“Not Risky”) to 3 (“Highly Risky”). This variable is well-suited to an ordered logistic analysis, formalized as follows:

2 $$\begin{aligned} Prob(Y_i \ge j) = \frac{1}{1 + exp(-\alpha _j - \beta _{1-6} \cdot \vec {X}_i + \beta _7 \cdot \tilde{R}_{i} + \beta _{8-9} \cdot \tilde{S}_i)} \end{aligned}$$

where Prob($Y_i \ge j$) is the probability of household *i* ranking livelihood *Y*’s riskiness above level *j* (where j $\in$ (“Not Risky”, “Somewhat Risky”)). The source of identification in this model is cross-sectional household variation. Specifically, the vector $\vec {X}_i$ represents demographic and geographic control variables that vary at the household level, denoted by *i*. Demographic variables include gender of head of household (with 1 representing a female respondent and 0 representing a male respondent), age, secondary school attainment (a binary variable in which 1 represents completing at least lower secondary school level), and household size. Geographic variables include (i) size of land operated and (ii) a binary variable reflecting whether a household is located within 1 km of one of the district’s two main rivers. $\vec {S}_i$ represents the information and social group index for household *i*. $\tilde{R}_i$ represents household *i*’s general climate risk perception. A potential omitted variable concern with this model is a respondent’s general level of worry; that is, households that generally have a high level of worry may lead to correlation between climate risk perceptions and overall livelihood risk perceptions. As a test for spurious relationships, we also include pension income among the set of livelihoods *Y*, which was perceived as the least risky livelihood category and is unlikely to be affected by climate risks.

We next seek to understand how household income sources are related to farmer climate risk perceptions, access to social and informational capital, and other demographic and geographic control variables. To do so, we construct a cross-sectional model in which we assess drivers of variation in households’ long-term income sources. This is specified as follows:

3 $$\begin{aligned} Y_{i}^k = \beta _0 + \vec {\beta }_{1-6} \cdot \vec {X}_i + \beta _7 \cdot \tilde{R}_i + \beta _{8-9} \cdot \tilde{S}_i + \epsilon _{i} \end{aligned}$$

In this model, $Y_{i}^k$ represents the proportion of household *i*’s income coming from livelihood *k* over the entire study period, 2015-2021. We assess four versions of this model for income coming from farming, livestock, off-farm employment, and migration remittances. The source of identification in this model is cross-sectional household variation, and control variables $\vec {X}_i$ are the same as in Eq. 2. We note that additional interaction effects are possible in the data generating process (e.g., the effect of perceiving high climate risks on household income diversification may be more pronounced for households with larger social networks), but the limited size of our sample prevents us from systematically investigating such effects.

Finally, we assess whether exposure to climate-related shocks significantly changes household income sources, both in the contemporaneous year of exposure and in subsequent years. We adjust Eq. 3 by introducing a time-varying dependent variable for household income sources and independent variables denoting exposure to droughts and floods:

4 $$\begin{aligned} Y_{i,t}^k = \beta _0 + \vec {\beta }_{1-6} \cdot \vec {X}_i + \beta _7 \cdot \tilde{R}_i + \beta _{8-9} \cdot \tilde{S}_i + \vec {\beta }_{10-11} \cdot \vec {H}_{i,t} + \delta _t + \epsilon _{i,t} \end{aligned}$$

**Fig. 3:**
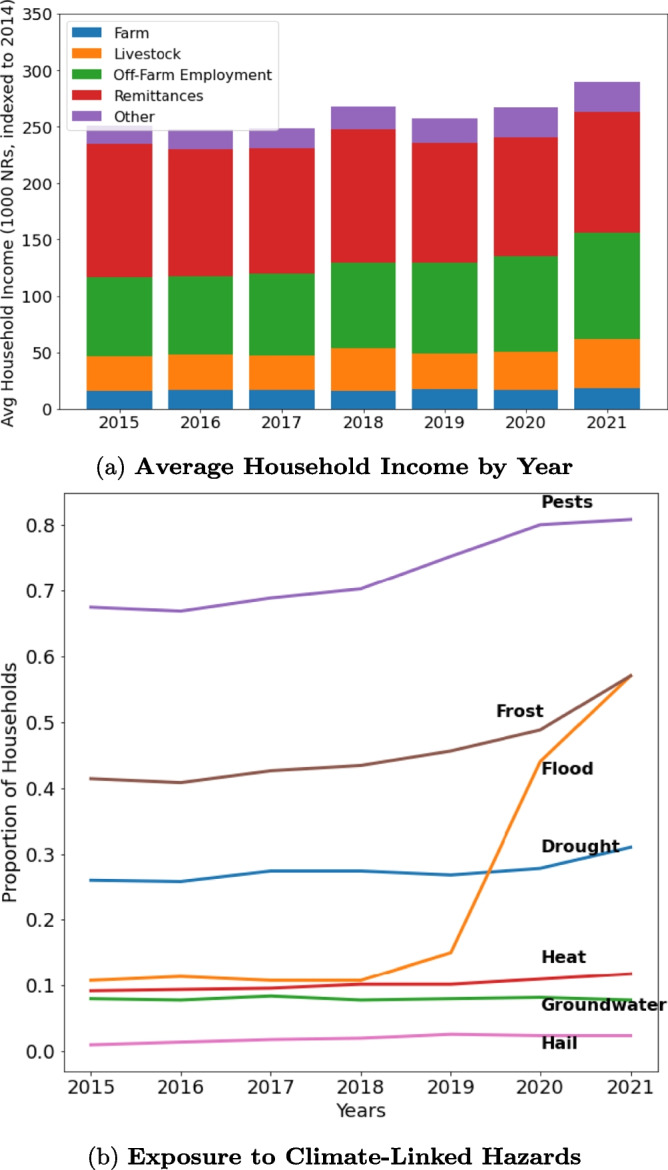
**Summary of household income sources and exposure to climate-linked hazards.** **a** Bar chart displays the average income composition of households from 2015 to 2021 by specific economic activity, expressed in thousands of 2014 Nepali rupees. On average, Chitwan farming households exhibit high diversity of economic activities, with farming only accounting for 8.8% of total reported income during this time. The most significant income-generating activities include: remittances from international migration (27.2%); off-farm employment, which includes wage labor (14.8%) and salary jobs (11.8%); and revenue from selling meat and milk (10.2%). Results are displayed for $N = 500$ observations in each year. **b** Line plots display proportion of households in our sample that reported exposure to each of seven climate-linked hazards in each year from 2015 to 2021. Of particular note, the proportion of households reporting exposure to floods increased substantially from 11% of respondents in 2015 to 57% of respondents in 2021. Results are displayed for $N = 500$ observations in each year

In this model, $Y_{i,t}^k$ represents the proportion of household *i*’s income coming from livelihood *k* in year *t*. $\vec {H}_{i,t}$ denotes whether household *i* reported exposure to a particular hazard in time *t*. For this analysis, we focus on exposure to floods and droughts as two hazards which a moderate proportion of the population reported experiencing in any given year (Fig. 3). Identification in this model comes from both household- and inter-annual variation in exposure to climate shocks. In alternate models, we test for the 1- and 2-year lagged effect of exposure to floods and droughts by replacing $\vec {H}_{i,t}$ with $\vec {H}_{i,t-1}$ and $\vec {H}_{i,t-2}$, respectively. Finally, we add time fixed effects ($\delta _t$) to control for any population-wide temporal trends in income sources. In Eqs. 3 and 4, we cluster errors at the ward level to account for potential heteroskedasticity imposed by our sampling strategy.

This model specification helps us address a potential source of endogeneity, in that farmers’ exposure to climate-driven risks is likely correlated with their geographic location (e.g., whether they live near or far from a river), which in turn is likely to be correlated with socio-economic variables (e.g., income, education, and caste identity). For example, as in many parts of the world, Indigenous groups native to the Chitwan region have historically been dispossessed of prime farming land and forced to move into more marginal land (Lipton & Bhattarai, 2014). While household resources and livelihood strategies are therefore likely to be correlated with long-term cumulative exposure to climate risks, we assume that a household’s exposure to floods and droughts in any specific year is randomly assigned. Further, we control for factors such as proximity to the region’s rivers and size of land operated as a proxies for long-term socio-economic factors that affect exposure to climate risks.

A further source of endogeneity could be that farmer perceptions of climate risks (our intervening variable) are themselves shaped by their dependence on different livelihoods (our dependent variable). For example, households that are more reliant on farming for income may feel more exposed to climatic risks than households that depend more on migration remittances. In SI 4.5, we examine other model specifications and test for possible endogeneity between our key independent variables and reliance on different livelihoods for income. We do not find strong evidence that household income composition is significantly correlated with generalized climate risk perceptions. However, we cannot fully discount the possibility that risk perceptions and livelihood income choices co-evolve, and an ideal research design could disentangle the direction of causality by collecting data on farming households’ risk perceptions over multiple survey waves.

## Results

Here, we present the most relevant summary statistics and econometric results for the models described in the “Econometric specifications” section. In the Discussion, we examine implications of these results for our formal hypotheses, underlying decision-making theories, and mechanisms driving environmentally induced poverty traps.

**Table 2:** Independent variable—summary statistics

Factor	Range	Mean	Std. Dev.	IQR
Hazards experienced at least once from 2015 to 2022	[0,7]	3.09	1.32	[2.00, 4.00]
Sources consulted at least once per year	[0,12]	3.93	2.17	[2.00, 5.00]
Groups participated in at least once per year	[0,5]	1.36	1.06	[1.00, 2.00]

Statistics are reported for $N = 500$ observations

### Descriptive statistics

Summary statistics for our main independent variables—hazard exposure, informational capital, and social capital—are reported in Table 2; the full list of hazards, information sources, and social groups, as well as their transformation into indices, are included in SI 2.1. Over the 7 year study period, households reported exposure to a mean of 3.1 different types of hazards and consulted an average of 3.9 different types of information sources while participating in 1.4 social groups at least once per year. As some hazards may exert greater influence on overall risk perceptions than others, and some information sources/groups may present more relevant information than others on local climate risks and livelihood opportunities, we present alternative specifications of these indices in SI 4. As expected, we observe substantial geographic variation in exposure to hazards, including droughts and floods (SI 3.4). Specifically, the mean number of years in which respondents reported a drought exposure ranged from 1.2 in Subward 2304 to 2.7 in Subward 2610; for flood exposure, this ranged from 0.8 in Subward 2304 to 2.7 in Subward 2611. Of particular note, we also observe a rapid increase in the number of households that reported exposure to floods across our sample during the course of the study period: whereas only 11% of respondents reported a flood in 2015, 57% reported such an exposure in 2021 (Fig. 3b). In general, this proportion aligns closely with proportions of Chitwan farmers in a recent survey who identified long-term declines in annual rainfall (56%) but higher intensity of rainfall (61%) (Thapa & Dhakal, 2024). Curiously, however, these proportions run counter to remotely observed trends in the region’s soil moisture as measured by the Standardized Evapotranspiration Index (SPEI). Such data indicate that the frequency of extreme drought or moisture incidents decreased over the course of the study period (SI 3.5). While SPEI measures suggest that extreme events in the region have declined in recent years, their geographic resolution (approximately 50 km by 50 km) is coarse and may not reflect localized experiences with floods, droughts, and other hazards. Further, SPEI values are subject to substantial sources of uncertainty, including the sample size of observations and the length of historical data used to calculate them, and may be prone to mis-classify extreme events (Laimighofer & Laaha, 2022). At the same time, individual respondents in our survey are likely to have different subjective thresholds of what constitutes a drought or flood. This variation likely introduces bias in measuring the Chitwan region’s overall exposure to climate-driven hazards. However, as our research questions concern how farming households perceive and respond to climate risks, we believe that an individualized, subjective measurement of exposure is most appropriate to our analysis.

**Table 3:** Intervening variables—summary statistics

Variable	Range	Mean	Std. Dev.	IQR
Risk direction index $\tilde{D}$	[-1,1]	0.407	0.395	[0.167, 0.667]
Salience index $\tilde{S}$	[0.169,1]	0.655	0.138	[0.610, 0.746]
Composite risk $\tilde{R}$	[$-$0.797,0.915]	0.268	0.270	[0.105, 0.455]

Statistics are reported for $N = 500$ observations

Our main intervening variable of interest is farmers’ perceived climate risk ($\tilde{R}$), which we measure along two dimensions: salience ($\tilde{S}$) and directionality ($\tilde{D}$). Summary statistics for each of these measures are displayed in Table 3 and Fig. 2. Generally, “long-term weather” rated the highest of all 14 factors regarding its impact on farmers’ economic success, with 74% of respondents assigning it a high importance (Fig. 2a). Furthermore, for most hazards, the majority of respondents assessed that the impact of climate hazards on their economic success was likely to worsen over the coming 5 years (Fig. 2b). This was especially true for pests, which 87% of farmers expect to worsen in the coming years. Farmers also largely expected floods (68% of respondents), frost (63%), and droughts (59%) to worsen. In general, our combined climate risk index ($\tilde{R}$), which could take on values between $-$1 and 1, ranged from a mean of 0.32 among respondents in Subward 2301 to 0.53 in Subward 2610 (SI 3.4).

**Fig. 4:**
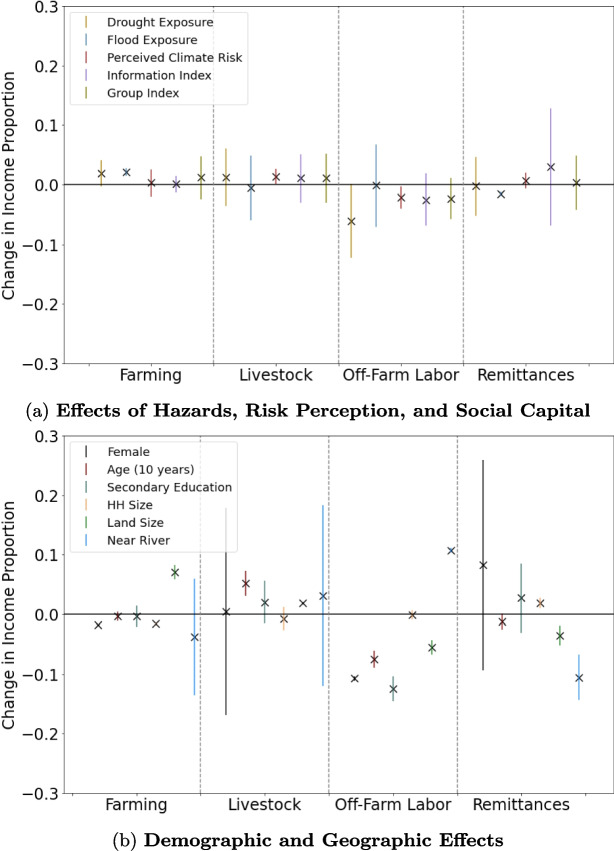
**Summary of effect sizes on household income proportion.** **a** Tick marks indicate the effect size of key independent variables on proportion of household income coming from (left-right) Farming, Livestock, Off-Farm Employment, and Remittances for the time series regression analysis (Eq. 3). Effect sizes and error bars representing the 95% confidence interval of each effect size are calculated for $N = 3427$ observations. **b** For the purposes of illustration, tick marks indicating effect sizes of demographic and geographic variables on the proportion of household income coming from each indicated livelihood are shown separately here. Non-binary variables (Perceived Climate Risk, Information Index, Group Index, Age, Household Size, and Land Size) are standardized to facilitate comparison

Our main outcome variable of interest in this analysis is households’ level of income diversification away from farming activities. In aggregate, households generally maintained highly diversified income portfolios throughout the survey calendar period; an average household derived 33.6% of its income from off-farm employment and 31.6% from migration remittances, with farming comprising only 8.8% of total income (Fig. 4a). However, income portfolios are highly variable across households: roughly 50% of respondents received less than 10% of their income from remittances, whereas 20% of households received 80% or more of their income from this source (SI 3.2). There are also significant temporal trends in the study area: average real household income (adjusted for inflation) increased by 15% over the study period, driven especially by growth in revenue from livestock (41% growth) and off-farm employment activities (35% growth, Fig. 4a). This may reflect increasing industrialization and agricultural commercialization in the Chitwan District, providing farming households with more nearby economic opportunities to diversify income sources.

### How salient are climate risk perceptions to general perceptions of livelihood risks?

Here, we investigate how farmer perceptions of climate risk may affect their general risk perceptions of four of the region’s main livelihood activities—farming cereal crops, raising livestock, engaging in off-farm employment, and migrating internationally—along with perceptions of pension income, which we believe should not be plausibly affected by climate risk perceptions. We indeed find that climate risk perceptions are not significantly associated with risk perceptions of pension income, which matches our intuition. However, we find that higher perceptions of climate risk are significantly and positively correlated with perceptions of livelihood risks for cereal crops, raising large animals, off-farm employment, and international migration (Table 4). In fact, climate risk perceptions are even more salient in driving general perceived risks of these latter three diversification strategies than they are to perceived risks of farming cereal crops. Specifically, an increase of 0.2 points on the Climate Composite Risk scale increases the odds that a household will assign a riskier ranking these livelihoods by 1.17, 1.22, 1.25, and 1.19 times, respectively ($p<0.01$). While we expect climate-linked hazards to drive higher risk perceptions of farming by adding uncertainty to harvests, these results suggest that farmers may also perceive rising climate threats to affect the viability of alternative livelihood strategies, either directly (e.g., by making migration trips or outdoor labor more hazardous), and/or indirectly by reducing households’ abilities to afford these options.

**Table 4:** General factors affecting risk perceptions of different livelihoods

Variable	Cereal crops	Large animal	Intl migration	Wage labor	Pension
Female	$-$0.0274 (0.220)	0.184 (0.211)	$-$0.368 (0.277)	0.215 (0.218)	0.333 (0.0216)
Age	0.0007 (0.009)	$-$0.0084 (0.008)	$-$0.0052 (0.011)	$-$0.0081 (0.009)	$-$0.0033 (0.009)
Secondary school	0.374$^{*}$ (0.223)	0.0462 (0.214)	0.397 (0.274)	$-$0.143 (0.225)	$-$0.0344 (0.218)
Household size	0.0234 (0.055)	0.0145 (0.053)	0.0545 (0.067)	0.0221 (0.055)	0.0389 (0.053)
Mean land operated	0.0115 (0.009)	0.0112 (0.009)	$$-$$0.0199$$^{**}$$ (0.010)	0.0072 (0.009)	$$-$$0.0041 (0.009)
Near river	$$-$$0.140 (0.238)	$$-$$0.0117 (0.225)	0.644$$^{**}$$ (0.272)	$$-$$0.0306 (0.236)	$$-$$0.211 (0.227)
Composite Climate Risk	0.160$$^{***}$$ (0.037)	0.201$$^{***}$$ (0.036)	0.227$$^{***}$$ (0.045)	0.177$$^{***}$$ (0.037)	0.0386 (0.036)
Information sources	$$-$$0.127 (0.096)	$$-$$0.0706 (0.093)	$$-$$0.0804 (0.113)	$$-0.292^{(**}$$ (0.094)	$$-0.197^{**}$$ (0.099)
Social networks	$$-0.187^{*}$$ (0.098)	$$-0.204^{**}$$ (0.096)	$$-$$0.199$$^*$$ (0.119)	0.0403 (0.100)	0.035 (0.099)

This analysis is conducted over $N = 498$ observations; two observations in which respondents gave the answer “don’t know” to questions about hazard risk and social contacts were discarded

Significance levels: $$^* p < 0.1$$, $$^{**} p<0.05$$, $$^{***}p<0.01$$

**Table 5:** Factors affecting household income composition—cross-sectional analysis

Variable	Farming	Livestock	Off-farm labor	Remittance
Constant	0.192$$^{***}$$ (0.014)	0.193$$^{***}$$ (0.048)	0.352$$^{***}$$ (0.023)	0.273$$^{***}$$ (0.073)
Female	$$-$$0.011$$^{**}$$ (0.004)	$$-$$0.0089 (0.090)	$$-$$0.0909$$^{***}$$ (0.008)	0.0752 (0.093)
Age	0.0007 (0.002)	0.0408$$^{**}$$ (0.019)	$$-$$0.0648$$^{***}$$ (0.012)	$$-$$0.0157$$^*$$ (0.008)
Secondary School	$$-$$0.0052 (0.005)	0.0128 (0.013)	$$-$$0.0971$$^{***}$$ (0.022)	0.0091 (0.038)
Household size	$$-$$0.0128$$^{***}$$ (0.000)	$$-$$0.0106 (0.008)	0.0019 (0.002)	0.0174$$^{**}$$ (0.007)
Land area operated	0.0588$$^{***}$$ (0.005)	0.0264$$^{***}$$ (0.004)	$$-$$0.0543$$^{***}$$ (0.003)	$$-$$0.0290$$^{***}$$ (0.010)
Near river	$$-$$0.0405 (0.037)	0.0309 (0.069)	0.102$$^{***}$$ (0.005)	$$-$$0.115$$^{***}$$ (0.016)
Composite climate risk ($$\tilde{R}$$)	0.0054 (0.011)	0.0121$$^{**}$$ (0.003)	$$-$$0.0259$$^{***}$$ (0.005)	0.0109 (0.008)
Social networks ($\tilde{G}$)	0.0134 (0.017)	0.0026 (0.023)	$-$0.0179 (0.020)	0.0057 (0.024)
Information sources ($\tilde{I}$)	$-$0.0039 (0.004)	0.0068 (0.023)	$-$0.0297 (0.025)	0.0391 (0.044)

Results from a series of ordinary least squares regressions on factors associated with household variation in the proportion of income coming from farming, livestock, off-farm labor, and remittances. All variables are household-level properties and are time-invariant in this model. This analysis is conducted over $N = 498$ observations; two observations in which respondents gave the answer “don’t know” to questions about hazard risk and social contacts were discarded

In SI 4.2, we further delineate the role of specific hazards in shaping general perceived risks of livelihoods. These results provide strong additional evidence that farmers perceive climate risks in multifaceted ways. While several climate-linked hazards (e.g., droughts, pests, and hail) are significantly associated with higher perceived risks of farming, as expected, some hazards (droughts, hail, and heat) are also correlated with higher perceived risks of common livelihood alternatives (e.g., livestock, migration, and off-farm employment). Similarly to our findings regarding the role of generalized climate risk perceptions, some of these specific hazards exert even stronger effects on the risk perception of common diversification strategies than they do on perceived farming risk. This complex relationship provides further support for psychological barriers to livelihood diversification. In addition to farmers’ aversion uncertain new technologies or livelihoods (Umar, 2014; Tessema et al., 2018), and the desire to reach a target harvest for food security or aspirational reasons (Bro, 2020; Lipion, 1968), multiple climate hazards are also contributing to farmers’ perceived risks of livelihood alternatives.

### What factors lead to household income diversification?

We next turn to an analysis of factors shaping household income diversification. Generally, we expected to find more diversified income portfolios for farmers perceiving higher climate risk (H1) and households with greater access to social and informational capital (H2), but less income diversification in years in which households experience a climate-related shock (H3). Our results indicate mixed support for these hypotheses. First, we find that climate risk perceptions are partially associated with long-term income portfolio strategies, but only for livestock and off-farm wage labor among the livelihood activities we investigate (Table 5). Further, this effect is somewhat opposite to H1: farmers perceiving higher climate risks generally depend more on livestock for income, and less on off-farm employment. While we anticipated greater reliance on livestock as a form of income diversification against climate risks, it is surprising that higher perceived climate risks are associated with less reliance on off-farm employment, whose risks are presumably less correlated to farming than livestock. Given the weak evidence for reverse causality (SI 4.5), this suggests that farmers may in fact be avoiding livelihood diversification strategies in response to climate hazards, which we further investigate in the next paragraph. Further, we do not find evidence for significant associations between access to social and/or informational capital and the composition of household income in our main specification, counter to our hypothesis H2. However, when measuring social capital by the number of friends with whom a respondent discusses economic matters (rather than participation in groups), we find that a higher degree of social capital is significantly associated with greater reliance on farming income, and decreased reliance on off-farm employment (SI 4.5). Again, the direction of this effect is counter to our hypothesis H2, which postulated that higher social and informational capital would be associated with a higher degree of livelihood diversification.

**Table 6:** Factors affecting household income composition

Variable	Farming	Livestock	Off-farm labor	Remittance
Constant	0.904$$^{**}$$ (0.41)	0.155 (0.525)	$$-$$0.374$$^{***}$$ (0.048)	0.707 (0.682)
Female	$$-$$0.0178$$^{***}$$ (0.001)	0.0049 (0.089)	$$-$$0.107$$^{***}$$ (0.002)	0.0828 (0.090)
Age	$$-$$0.00365 (0.004)	0.052$$^{***}$$ (0.011)	$$-$$0.0751$$^{***}$$ (0.007)	$$-$$0.0123$$*$$ (0.007)
Secondary school	$$-$$0.0035 (0.009)	0.0201 (0.018)	$$-$$0.125$$^{***}$$ (0.011)	0.0273 (0.029)
Household size	$$-$$0.0160$$^{***}$$ (0.001)	$$-$$0.0074 (0.010)	$$-$$0.0010 (0.004)	0.0188$$^{***}$$ (0.005)
Land area operated	0.0705$$^{***}$$ (0.006)	0.0187$$^{***}$$ (0.000)	$$-$$0.0554$$^{***}$$ (0.006)	$$-$$0.0351$$^{***}$$ (0.009)
Near river	$$-$$0.0381 (0.050)	0.0313 (0.078)	0.107$$^{***}$$ (0.002)	$$-$$0.106$$^{***}$$ (0.019)
Composite climate risk ($$\tilde{R}$$)	0.0029 (0.011)	0.0138$$^{**}$$ (0.006)	$$-$$0.0215$$^{**}$$ (0.009)	0.0065 (0.007)
Social networks ($\tilde{G}$)	0.0118 (0.018)	0.0109 (0.021)	$-$0.0228 (0.018)	0.0037 (0.023)
Information sources ($\tilde{I}$)	0.0009 (0.007)	0.0109 (0.020)	$-$0.0249 (0.022)	0.0298 (0.050)
Flood exposure	0.0209$$^{***}$$ (0.003)	$$-$$0.0054 (0.028)	$$-$$0.0011 (0.035)	$$-$$0.0152$$^{***}$$ (0.002)
Drought exposure	0.0191$^*$ (0.011)	0.0126 (0.025)	$-$0.0609$^*$ (0.032)	$-$0.0028 (0.025)
Year	$$-$$0.0089 (0.006)	0.0002 (0.006)	0.0103$$^{***}$$ (0.001)	$$-$$0.0068 (0.008)

Results from a series of ordinary least squares regressions on factors associated with changes in the proportion of income coming from farming, livestock, off-farm labor, and remittances. Dependent variables, along with the flood and drought exposure variables, are expressed at the household-year scale. Other variables are household-level properties and are time-invariant in this model. The variable year represents annual fixed effects to capture community-scale temporal trends in livelihood incomes. This analysis is conducted over $N = 3427$ observations, reflecting 7 annual observations for each of 500 households. 73 household-year observations in which respondents answered “Don’t Know” for a climate exposure and/or income source in a particular year were dropped from the analysis

By investigating household livelihood responses to inter-annual variation in exposure to shocks, we can develop further understanding of the drivers of livelihood diversification or intensification. Our time-varying econometric specification indicates that experiencing climate-driven events, particularly floods, is significantly associated with increased reliance on farming income (Table 6), providing support for hypothesis H3. Specifically, exposure to a flood is significantly associated with a 2.1 percentage point increase in the proportion of household income coming from farming ($p < 0.01$), and exposure to drought is marginally associated with a similar increase of 1.9 percentage points ($p < 0.1$). These events also are associated with decreased reliance on different types of income diversification strategies: exposure to floods is associated with a 1.5 percentage point decrease in reliance on migration remittances ($p<0.01$), while exposure to drought is marginally associated with a 6.1 percentage point decrease in the proportion of household income coming from off-farm employment ($p < 0.1$). These findings are consistent with several studies across the developing world that find a correlation between farmers’ exposure to shocks and diminished livelihood diversification. For example, farmers in China also exhibited increased income reliance on maize farming in response to both increased precipitation and drought conditions (Ma & Maystadt, 2017), while Ugandan farmers experiencing recent droughts or floods were less likely to exhibit income diversification (Antonelli et al., 2022). Further, household exposure to flood damage in Indonesia was correlated with decreased likelihood of permanent migration (Bohra-Mishra et al., 2014), and Bangladeshi households experiencing severe crop failure were less likely to migrate either locally or internationally (Gray & Mueller, 2012), while Nepali households perceiving environmental degradation were more likely to shift from international to local migration (Massey et al., 2010).

**Fig. 5:**
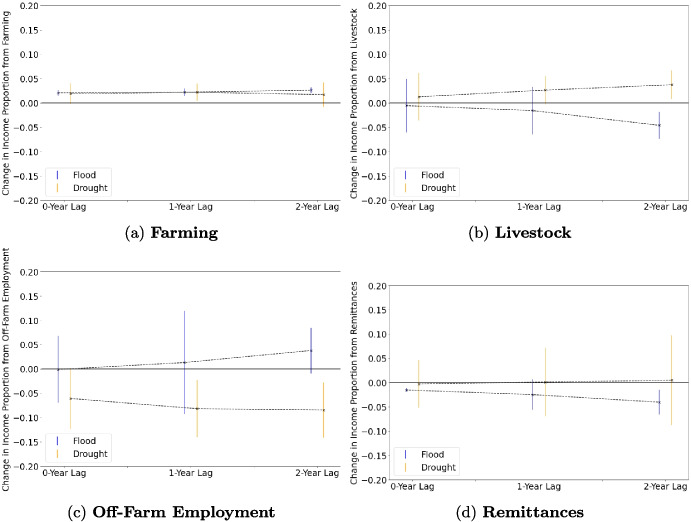
**Size and significance of exposure to climate-linked hazards over 2-year period.** The effect size of exposure to drought (orange) and flood (blue) on the proportion of income coming from **a-d)** farming, livestock, off-farm employment, and remittances are shown for three time periods: income in the same year a household reports exposure to the event ($N = 3427$ observations), income from 1 year after ($N = 2929$ observations), and income from 2 years after experiencing the event ($N = 2431$ observations). In some cases (e.g., livestock and off-farm employment), exposure to a hazard is associated with an even greater effect on income proportions 2 years after the event. Error bars represent the 95% confidence interval

**Fig. 6:**
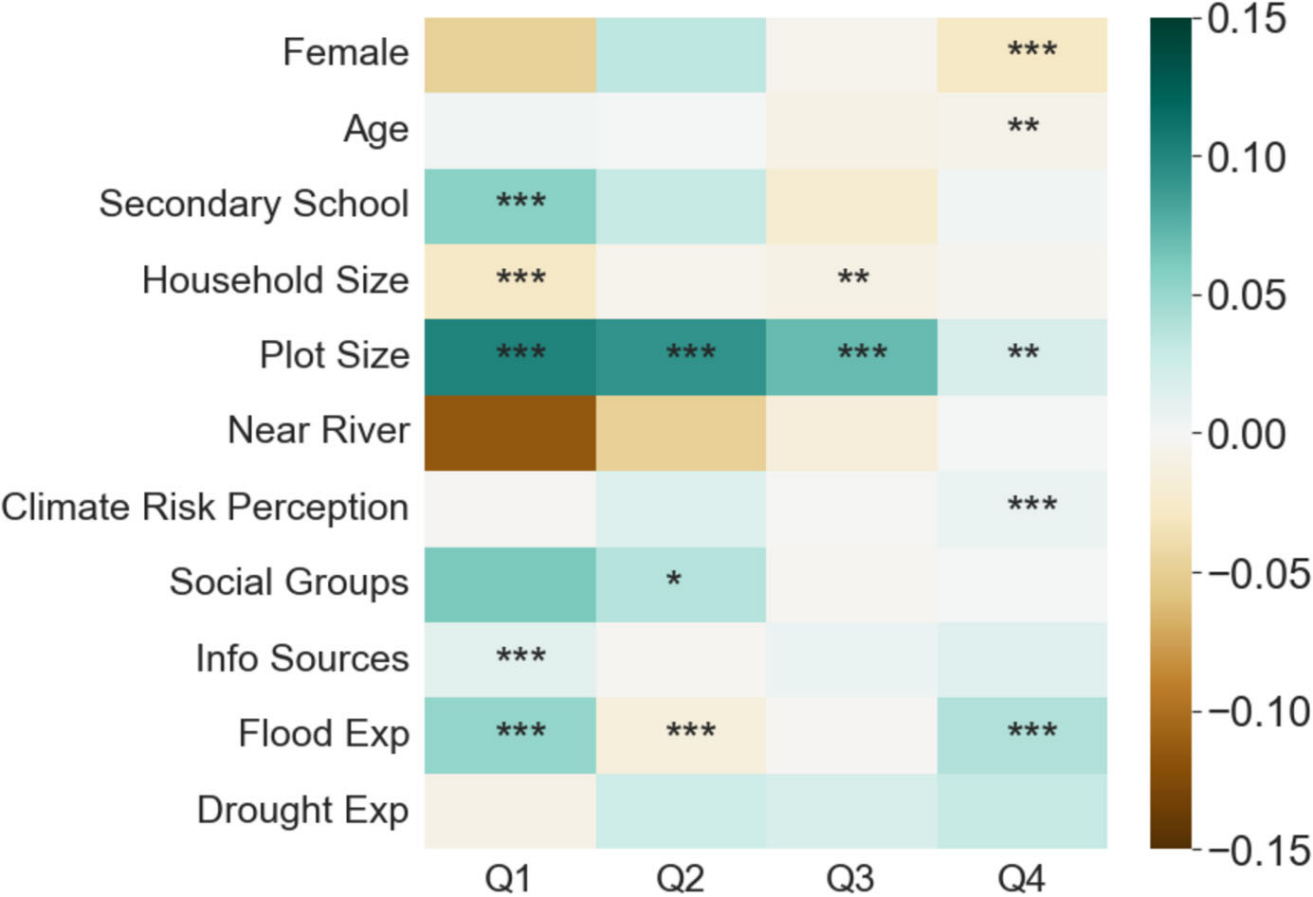
**Effect sizes and significance on income proportion by quartiles.** Heatmaps display the direction (blue = positive, brown = negative), strength (colorbar), and significance ($$^* p < 0.1$$, $$^{**} p < 0.05$$, $$^{***} p < 0.01$$) of effects of each variable on the y-axis on the proportion of household income coming from farming activities. Columns represent effects disaggregated for each income quartile, with Q1 representing the lowest income quartile, and Q4 the highest quartile. As quartiles were calculated based on respondents’ mean annual income, quartiles have different numbers of observations depending on whether a respondent answered “Don’t Know” to a question about income or exposure in a given year. Specifically, the number of observations from Q1 to Q4, respectively, are $$N =$$ 911; 1007; 812; and 697. Similar results for income from livestock, off-farm employment, and migration remittances are shown in SI 4.6

One driver of these results could be short-term adaptation responses: for example, households may be bringing migrants and laborers back to the farm to manage clean-up and salvage harvests after an extreme event (Gray & Mueller, 2012). However, in our study, these effects also appear to persist or even increase in magnitude in subsequent years after a household experiences such a shock (Fig. 5). For example, exposure to flood is associated with significant increases in the proportion of income coming from farming and significantly less reliance on migration remittances even 2 years after experiencing such an event, with more substantial effects in the years following an exposure. Meanwhile, exposure to drought is associated with an even more substantial decrease in the income proportion coming from wage labor in the 2 years following the event. By contrast, households experiencing drought are more likely to rely on raising livestock 2 years after exposure. Each of these results suggest that households may also be adjusting longer-term livelihood strategies—generally increasing their reliance on farming—in response to experiencing a climate-driven extreme event.

Our panel model therefore points to a potential poverty trap: exposure to natural hazards that would clearly affect the viability of crop yields actually further deepens farmers’ reliance on this livelihood for income in both the immediate and long term. This could be driven by at least two mechanisms: (i) financial constraints (i.e., a natural hazard-driven shock reduces households’ disposable incomes, and thus their ability to diversify livelihoods in a given season) and/or (ii) psychological responses (i.e., a shock induces households to “hunker down” and focus even more financial and labor resources to produce a suitable harvest). We attempt to further disentangle these mechanisms by disaggregating results by income quartiles. If financial constraints were the dominant mechanism driving an increased reliance on farming income during hazards, we would expect this effect to be more pronounced in lower quartiles and less pronounced in higher quartiles. By contrast, if psychological responses drove this behavior, we would expect little difference in effect across quartiles, and perhaps even more of a retrenchment effect among farmers in higher quartiles, who may have more resources to re-deploy to farming in a given season. This is especially true if farmers’ primary objective is to meet a harvest aspiration that is threatened by a climate-linked hazard (Umar, 2014), and/or if climate risks also make diversification options even riskier than farming.

Our results illustrate quartile-specific effects that differ by hazard (Fig. 6). Specifically, exposure to floods is associated with an especially strong reliance on farming income among the lowest income quartile, increasing this proportion by 5.1 percentage points ($p < 0.01$). However, exposure to floods is also associated with a significant increased reliance on farming for the highest-income quartile farmers (3.9 percentage points, $p<0.01$). Curiously, we do not observe this doubling down phenomenon among the middle quartiles; there is no significant effect of flood exposure on farm income reliance among the second-highest quartile, and the second-lowest income quartile actually exhibits a small but significant diversification away from farming ($-$1.4 percentage points, $p<0.01$). Though these quartile-specific results by themselves do not provide definitive evidence of a particular mechanism, the observation that a “doubling down” effect on farming occurs at the two income extremes suggests that there may be both financial (for the lowest quartile) and psychological (for the highest quartile) mechanisms at play.

Additional quartile-specific results also suggest further insights. Education and access to information sources exhibit significant positive correlations with reliance on farming among the lowest income quartile, but are not significantly correlated with the farming income proportion for higher-income quartiles. Perhaps most tellingly, the size of land farmed exhibits a significant, positive correlation on the household farming income proportion across all quartiles, but the size and significance of this effect decreases steadily from the lowest (10.2 percentage points, $p<0.01$) to highest-income quartile (1.9 percentage points, $p<0.05$). Conversely, farmers’ climate risk perception exhibits a small but significant correlation with increased reliance on farming income among the highest-income farmers, but is not significant for other quartiles. This provides further evidence that financial, physical, and informational capital constraints may be key determinants of livelihood strategies for lower-income households, whereas higher-income households are less constrained by such factors, and more likely to adjust livelihood strategies based on risk perceptions.

## Discussion

While previous work has elaborated various factors that impact farmers’ climate risk perceptions and on-farm adaptation choices, here, we investigate the role of climate-linked hazards and risk perceptions in shaping farmers’ evaluation of a range of livelihood strategies. We derive three main conclusions from our analysis. First, climatic conditions appear to be highly salient to farmers’ overall perceptions of livelihood risks, including farming and non-farming occupations (“Descriptive statistics”). Further, climate-related risks appear to drive even higher perceived risks of common alternative livelihood options to farming (e.g., international migration and off-farm employment) (“How salient are climate risk perceptions to general perceptions of livelihood risks?”). Finally, we find that even when households perceive high climate risks, they may in fact further retrench into farming-based activities during periods of acute climate shocks (“What factors lead to household income diversification?”). This behavior persists across multiple years and appears to be driven by a complex suite mechanisms: financial constraints that impede lower-income households from quickly diversifying income sources, a psychological desire among farming households to avoid harvest losses, and heightened risk perceptions of livelihood alternatives due in part to climate factors.

**Table 7:** Theoretical frameworks—application to smallholder farmer climate adaptation

Framework	Relevanthypotheses	Supporting evidence	Contradicting evidence
Protection Motivation Theory	H1	Climate hazards correlated with perceived livelihood risks	Climate risk perceptions not correlated with income diversification
New Economics of Labor Migration	H2	Households maintain diversified income portfolios	Social capital not correlated with diversification
Security-Potential/Aspiration	H3	Farmers depend more on farming during shocks	Retrenchment into farming may reflect risk aversion to livelihood alternatives

Our findings provide additional nuance for the three main theoretical frameworks that informed our hypotheses (Table 7). On the one hand, we find that over long time scales, rural households in Chitwan generally maintain diversified income portfolios, in line with NELM. However, neither higher perceived climate risks nor greater access to informational and social capital appear to drive income diversification, counter to predictions from NELM and PMT. On the other hand, in times of acute income shock, rural households tend to intensify their farming livelihoods—providing more support for the SP/A framework, which emphasizes the goal of meeting a basic aspiration level. These nuances align with recent findings on drivers of income diversification across a variety of subsistence farming contexts. While farmers that experience sustained and severe climate-linked hazards appear more likely to diversify income portfolios (Ma & Maystadt, 2017; Wuepper et al., 2018; Arslan et al., 2021; Antonelli et al., 2022), households experiencing short-term, anomalous shocks are likely to respond through intensifying current agricultural practices (Ma & Maystadt, 2017; Antonelli et al., 2022). Further, households that already maintain highly diversified portfolios are generally less likely to further adjust income sources in response to a climate-linked shock (Arslan et al., 2021). Our findings contribute to this literature by identifying the role of climate-driven hazards in heightening risk perceptions of livelihood alternatives as another factor that contributes to divergent outcomes on diversification. As such, the retrenchment response to climate risks may in fact reflect risk-averse behavior, and not the loss-averse, risk-seeking behavior predicted by SP/A.

More broadly, our findings also suggest additional mechanisms by which rural households in developing contexts may be engaged in poverty traps. Previous literature has elaborated several such mechanisms, including feedbacks between poverty, under-nutrition, and diminished human productivity (Dasgupta, 1998); shortened decision-making periods among individuals in poor and/or volatile contexts (Barrett & Carter, 2013); and aversion to perceived risks of livelihood alternatives (Bryan et al., 2014). To complement this literature, we elaborate additional mechanisms through which climate risks may contribute to poverty traps. In the short term, exposure to climate-linked hazards (e.g., droughts or floods) may induce a retrenchment response in which rural households increase their reliance on farming for income, and decrease their reliance on alternative livelihoods (e.g., off-farm employment and international migration). In our analysis, we find evidence for hypothesized financial mechanisms (i.e., hazards may erode poor farming households’ financial, physical, and human resources to deploy alternative strategies to farming) and psychological mechanisms (i.e., households’ primary objective is to meet a harvest aspiration, and even wealthy households will reduce investment in alternatives if this is under threat). Over the longer term, smallholder farmer perceptions of increasing climate risks may also increase their risk perceptions of alternative or complementary livelihoods to farming, including off-farm employment and international migration. Consequently, rural households may deepen their reliance on subsistence farming, which is becoming increasingly threatened by these same climate-driven risks, further eroding assets to escape poverty.

The contribution of climate risks to poverty traps represents a potentially significant impediment to development policy goals at local and global scales. For example, Nepal’s Agriculture Development Strategy seeks to reduce rural poverty from 27 to 10% by 2035, largely through commercialization of the agricultural sector and diversification of rural livelihoods to service and industrial sectors (Nepal Ministry of Agricultural Development, 2015). However, these goals are likely to be delayed if increasingly severe climate risks elicit further retrenchment into subsistence farming activities. At the global level, the World Meteorological Organization has developed a Global Framework for Climate Services to enhance access to climate information in order to improve management of climate risks, particularly among the most vulnerable (World Meteorological Organization, 2014). Yet, while the technical capacity to collect climate data has improved in many parts of the world, providers of climate information services still lack sufficient understanding of the decision contexts in which vulnerable populations might use this information (Findlater et al., 2021). In particular, climate information services for smallholder farmers generally have a myopic focus on agricultural impacts, ignoring the implications of climate risks on other livelihood options farming households may pursue.

Our findings lead to insights that could help improve the effectiveness of such efforts. First, investments in expanding access to climate information services should be paired with financial resources that provide low-income farmers with improved capability to diversify livelihoods. Previous research has demonstrated that government investments in disseminating climate information has often had limited effect on promoting farmer livelihood diversification (Ziervogel, 2004), and in some cases may provide recipients with a greater sense of security (Dang et al., 2014). In our study, we find that access to greater informational capital is associated with an increased reliance on farming among respondents in the lowest income quartile, arguably a demographic for which diversification is most crucial to surviving climate hazards. Policy packages that pair dissemination of climate information with subsidized crop insurance, cash transfers, or migration assistance may be more effective in encouraging livelihood diversification (Choquette-Levy et al., 2021). Second, policymakers should take a broader view of climate information services such that these provide accurate information about risks to livelihood alternatives, and ideally point to less risky opportunities to diversify incomes during shocks. Specifically, climate information services should focus not just on risks to farming crops, but also on climate risks to livestock, off-farm employment, and rural–urban migration, among other livelihood diversification options. Our results point to a strong correlation between farmers who are concerned about climate change and who believe that livelihood alternatives are also highly risky. These perceptions may reflect real risks, including heat stress that makes off-farm labor work more dangerous and extreme events that make migration trips perilous or less profitable. Third, officials can consider deepening investments in mechanisms that spread crop yield risks over multiple harvests and/or reduce yield volatility (e.g., irrigation infrastructure, cooperatives, grain silos, and food banks). Although policy approaches to agricultural climate risks often assume that farmers will self-insure through migration and other forms of livelihood diversification, our findings of retrenchment behavior during climate-linked shocks suggest a desire to maintain harvests. Therefore, risk-sharing mechanisms tailored to the agricultural sector may provide rural households with both financial and psychological security to pursue alternative income-generating activities. However, such mechanisms may become less effective over time, if rising climate risks lead to increasingly correlated losses across households and across seasons.

There are several avenues for future study that could further improve theoretical development around a retrenchment response to climate risks. Collecting data on farming households’ risk perceptions over multiple survey waves could further disentangle the direction of causality between perception of climate risk and reliance on different livelihoods for income. From an analytical perspective, it would be fruitful to integrate data from mostly close-ended survey questions with qualitative insights from in-depth interviews and focus group discussions. Questions on how farmers compare risks across different livelihood options and the type of information obtained from different sources would be especially relevant to this analysis. Expanding data collection to different agro-ecological regions of Nepal, particularly farming areas in the Himalaya and mid-Hills, could also provide useful insights on how different types and degrees of climate risks are shaping farmers’ livelihood choices. Finally, further exploring rural households’ basic aspirations and how these might be altered by an income shock (e.g., a drought or flood) may facilitate more nuanced theoretical development.

Nevertheless, our findings provide insight on how climate risks introduce new barriers to livelihood diversification among smallholder farmers, which may deepen poverty traps and threaten development goals as such risks intensify. In particular, the role of climate-linked hazards in exacerbating risk perceptions of alternative livelihoods to farming is an under-studied mechanism that may partially explain the slow livelihood diversification response in regions that are highly exposed to such hazards. This highlights the need for more comprehensive policy approaches that address both financial and psychological needs of climate-vulnerable populations in order to provide viable options for managing such risks.

## Supplementary Information

Below is the link to the electronic supplementary material.

Supplementary file 1 (pdf 3228 KB)

## Data Availability

All de-identified data and analysis code used for this manuscript is currently available via the following public GitHub repository: https://github.com/nchoquettelevy/Nepal_Climate_Survey. At the time of submission, we have started the process to transfer the data to a public repository via the ICPSR Consortium.

## Footnotes

1 Our sample size of 500 respondents provides sufficient power (0.80) at a significance level of $\alpha = 0.05$ to detect a standardized effect size of 0.25 in variation in a dependent variable (e.g., perceived climate risk, income source) due to variation in an independent variable (e.g., exposure to a climate hazard, gender, information sources). This threshold is consistent with similar standardized effects of 0.2–0.3 detected in other studies of climate risk perceptions due to variation in geographic and demographic variables (Dang et al., 2014; Buchanan et al., 2019) which used comparable sample sizes ($N = 600$ and $N = 462$, respectively). However, the sample size limits our ability to reliably detect significant interaction effects between multiple independent variables.
