# Supplementary material for: Retrenchment under climate-driven risks in subsistence farming communities
Source: Popul Environ. 2025 May 20;47(2):22. doi: 10.1007/s11111-025-00493-8 (PMC12092560; doi:10.1007/s11111-025-00493-8)
Supplement: Supplementary file 1 — (pdf 3228 KB) [file 11111_2025_493_MOESM1_ESM.pdf]

Supplemental Information: Retrenchment under Climate-Driven Risks in Subsistence Farming Communities

1 Theoretical Framework and Hypotheses

In addressing our research questions, this study tests hypotheses emerging from three theoretical frameworks that are especially relevant to questions of how subsistence farming households perceive and act on climate risk: Protection Motivation Theory (PMT), New Economics of Labor Migration (NELM), and Security Potential/Aspiration (SP/A). Protection Motivation Theory (PMT) states that decision-makers generally seek to mitigate the risk of perceived threats [2, 3, 9]. The degree to which they act in accordance with this principle depends on two main variables: the perceived severity of a threat and the perceived capacity to mitigate this risk. A more generalized framework, the Theory of Planned Behavior (TPB), also frames individuals' actions in terms of their attitudes towards a behavior and their perceived control in carrying out that behavior, and also adds that social norms shape how strongly an individual considers a behavior [1]. Therefore, we should expect that the more farming households perceive climate change as a threat, and/or the more that households believe they have sufficient resources to mitigate climate hazards, the more likely they should be to take observable forms of climate adaptation, including livelihood diversification [4]. We hypothesize that households with recent experience with climate hazards are more likely to perceive climate change as a salient threat to their livelihoods. Furthermore, access to diverse sources of information is likely to increase the perceived severity of climate change, as well-informed households are more likely to hear of and understand climate information. This should especially be true for households that are connected with professionalized information sources, e.g. scientists and agricultural extension officers, who are likely to be more knowledgeable about climate risks than the general public.

A second relevant theory comes from the New Economics of Labor Migration (NELM), which postulates that households engaged in rural livelihoods seek to minimize risks to their livelihoods and overcome constraints in access to financial capital [10]. Additionally, households aim to minimize their sense of relative deprivation (i.e., a perceived gap between their well-being and that of others in their social network) [8]. A common strategy to cope with risk is to diversify sources of household income by engaging in multiple livelihood strategies, including rural-urban migration [6]. Based on these principles, if households perceive elevated risks to their livelihoods due to climate change, especially in emerging market contexts with limited access to financial services, then they should be more likely to diversify their livelihood portfolios. Furthermore, we hypothesize that households with larger social networks are more likely to feel a sense of relative deprivation compared to members of their network who may be engaged in alternative livelihoods. This is especially true if farmers are part of social groups that encompass peers living in different geographic locations or engaged in different economic activities (e.g., migrant groups and money lending circles). Conversely, households with smaller social networks may be less motivated to change livelihood strategies. A similar hypothesis follows from PMT: in contexts with limited access to formal institutions, households belonging to social groups (e.g., farmer cooperatives and women's groups) are more likely to believe they have the financial and social capital to address climate risks. A potentially countervailing mechanism is that households enmeshed

in rich social networks, particularly those focused on local agriculture (e.g.,farmer cooperatives), may be subject to a social norm to resist diversifying away from farming livelihoods. Our next set of formalized hypotheses are thus:

While NELM and PMT provide testable implications for whether farming households take observable actions to reduce climate risk, they do not differentiate between the different risk management options that farmers may choose from. By contrast, Cumulative Prospect Theory (CPT) [12] and a related framework, Security-Potential/Aspiration (SP/A) [5], provide more granular theory on how decision-makers choose among a portfolio of risky options. Both frameworks start with the assumption that decision-makers interpret risky outcomes as either gains or losses relative to a subjective reference point. In both frameworks, individuals also exhibit risk aversion (i.e., decreasing marginal utility of gains) and loss aversion (i.e., losses are penalized with greater weight than gains of an equivalent magnitude). One additional component of SP/A is that decisions are framed as a dual-objective process: individuals assess their potential gains or losses relative to an aspiration (similar to the reference point in CPT), but may differ in terms of how much utility weight they assign to the worst possible outcomes (the security potential). If those outcomes are unlikely to meet a basic aspiration level, a security-minded individual may become risk-seeking in order to minimize or eliminate a loss. If the aspiration level has a good chance of being met, the individual then becomes risk-averse in evaluating additional gains. This framework may have particular relevance for farmers evaluating multiple adaptation options in light of climate shocks that threaten their basic subsistence. Specifically, when exposed to a shock (e.g., a drought or flood), smallholder farmers may be expected to choose riskier adaptation strategies that have the potential to minimize or eliminate their losses, whereas in the absence of climate shocks, farmers may be expected to choose less risky strategies.

Table 1 summarizes the basic decision-making objectives, relevant factors, and limitations in applying each theoretical framework to smallholder farmer climate adaptation. While they focus on different scales and aspects of decision-making under uncertainty, all three frameworks broadly predict that farmers should mitigate climate risks to their livelihoods to various degrees. NELM and PMT also suggest that greater access to social and informational capital should be correlated with a higher propensity to diversify livelihoods, both because these networks can highlight the salience of climate risks, and also because they may give farmers more confidence in their ability to implement an action. However, the PMT framework is unique in highlighting the salience of perceived climate threat as an important factor in decision-making, and SP/A uniquely focuses on the role of farmers' aspiration levels and security weighting in determining their choices among risky options.

**Framework**	**Decision Objectives**	**Relevant Factors**	**Limitations**
New Economics of Labor Migration	Minimize livelihood risk; Minimize sense of "relative deprivation"; Overcome credit constraints	Perceived income volatility; Comparison to social networks	Doesn't distinguish between adaptation options
Protection Motivation Theory	Mitigate risk of perceived threats	Perceived threat severity; Perceived capacity to mitigate threat; Social norms	Only applicable to perceived threats
Security-Potential /Aspiration	Meet basic aspiration level	Aspiration target; Security of meeting aspiration	Difficult to ascertain aspiration targets

Table 1: Theoretical Frameworks - Application to Smallholder Farmer Climate Adaptation

2 Operationalizing Variables

This section provides supplementary detail to Section 2.3 in the main text on how key variables were coded and operationalized for analysis.

2.1 Independent Variables

Respondents were asked to indicate their exposure to the hazards, information sources, and social groups listed in Table 2 below. First, respondents were asked to recall if each of the specific hazards listed in Table 2 had affected their crops and harvests in each year from 2015-2021. Then, respondents were asked to identify how frequently they consulted each of the items listed in the Information Sources column ("Never", "Less than once per month", "Once per month", "2-3 times per month", "Once or more a week", "Don't know) and to indicate their level of trust in each source ("Little or no trust", "Some trust", "Mostly or completely trust", "Don't know"). Finally, respondents indicated whether they participated in each of the groups listed in the last column of Table 2, and if so, how many meetings of the group they had attended over the past year. These responses were used to construct the summary statistics in the main text Section 3.2.

**Hazards**	**Information Sources**	**Social Groups**
Drought	Radio	Women's group
Flood or heavy rain	Television	Youth group
Lack of groundwater	Newspapers	Farming cooperative
Excess heat	Agricultural Office	Livestock group
Pests	Veterinarian	Migrants from Chitwan
Frost	Migrant Labor Agency	Community Forest User Group
Hail	Other government source	Caste or jati-based lending group
	Relative or friend in village	Other
	Relative or friend outside of village	
	Religious authority	
	Scientist	
	Social media<br>(e.g., Facebook, Messenger, WhatsApp)	
	Other	

Table 2: List of Specific Event Frequency Questions

To facilitate analysis, we also create standardized indices for each of these variables. In general, these indices were constructed as:

$$\tilde{I}_i^k = \frac{I_i^k - \bar{I}^k}{\sigma_{I^k}} \tag{1}$$

where $\tilde{I}_i^k$ represents the standardized index value for household i for variable k (where k is the set [Hazards, Groups, Information Sources]). I_i^k represents the raw count of the index. For hazards, this represents the number of hazards listed in Table 2 for which the respondent indicated at least one exposure over the 7 previous years. For information sources, this represents the number of sources in Table 2 which the respondent consults at least once per year. Similarly for social groups, this represents the number of groups in Table 2 in which the respondent or members of his/her household participated at least once in the past year. $\bar{I}^k$ represents the mean number of hazards, groups, or sources counted in this fashion across the survey population. The factor σ_I^k represents the standard deviation of I^k across the survey population for variable k. The standardized index $\tilde{I}_i^k$ thus represents the number of standard deviations that household i is above (positive) or below (negative) the survey

sample mean with respect to its exposure, access to information sources, and participation in social groups. Alternative specifications of these variables, including decomposition by principal components, are presented in SI 4.

2.2 Climate Risk Perceptions

To facilitate the construction of a composite climate risk index, $\tilde{R}_i$ (main text, Section 2.2), we combine a directional measure of whether a respondent generally believes climate impacts to be worsening, improving, or remaining about the same ($\tilde{P}_i$) and a measure of the overall salience of climate risks ($\tilde{S}_i$) as follows:

$$\tilde{R}_i = \tilde{P}_i * \tilde{S}_i \tag{2}$$

such that $\tilde{R}_i$ takes on values in the interval [-1,1], with -1 representing a farmer to whom climate is a highly salient risk factor, but that climate-driven hazards are likely to alleviate in impact over the coming years; and +1 representing a farmer who believes climate is highly salient and that these factors are likely to worsen in the coming years.

The first dimension, $\tilde{P}_i$, was assessed by asking respondents to rate the impact of each of the hazards listed in Table 2 on crop harvests over the next 5 years ("Less Severe", "No Significant Change", "More Severe", or "Don't Know). A generalized directional risk index, $\tilde{P}_i$, was calculated based on these responses as:

$$\tilde{P}_i = \frac{\sum_{h=1}^{h=7} P_{i,h}}{7} \tag{3}$$

where $P_{i,h}$ represents respondent i's perception of the future risk for hazard h, and takes the value -1 if rated as "Less Severe", 0 if rated as "No Significant Change" or "Don't Know", and 1 if rated as "More Severe". $P_{i,h}$ therefore ranges from -1 (respondent believes all hazards are likely to become less severe in the coming years) to +1 (respondent believes all hazards are likely to become more severe in the coming years).

The salience of climate risks ($\tilde{S}_i$) was assessed by asking respondents to rate the impact of each of the risk factors listed in Table 3 on their economic success ("No influence" = 1, "Some influence" = 2, "A lot of influence" = 3, "Don't Know" = Not rated). The relative salience of climatic factors in respondents' decision-making, S_i, was calculated as follows:

$$S_i = L_i^c - \bar{L}_i^{k \neq c} \tag{4}$$

where L_j^k represents the Likert score assigned by household j to factor k, and c represents the long-term weather factor specifically. This measure distinguishes the degree to which long-term weather factors are seen as influential to economic success from farming, relative to other factors.

We standardize $\tilde{S}_i$ to take on only non-negative values, such that the directional value of $\tilde{R}_i$ reflects whether a respondent believes climate impacts to be worsening (positive values of $\tilde{P}_i$ and $\tilde{R}_i$) or improving (negative values of both). In either case, $\tilde{S}_i$ affects the absolute value of $\tilde{R}_i$ without affecting its direction. As the raw salience measure S_i (main text, Equation 2) can take values between -2 and 2 (as the lowest possible value of S_i is $1 - 3 = -2$, and highest possible value of S_i is $3 - 1 = 2$), we translate the variable by 2 and then standardize to the maximum observed value:

$$\tilde{S}_i = \frac{S_i + 2}{S_{i,max} + 2} \tag{5}$$

Risk Factors
Access to Farm Labor
Health of respondent and family
Access to Farm Technology
Access to Financial Resource
Education of respondent and family
Quality of seeds
Quality of soil
Water availability
Size of land
Short-term weather conditions < 2 weeks
Long-term weather conditions > 2 weeks
Pests
Access to commercial markets
Economic conditions of Chitwan
Religious Factors
Other

Table 3: List of Risk Factor and Livelihood Options

2.3 Livelihood Diversification

Table 4 details specific livelihood options included in the survey, and the broader categories to which they were coded. For each specific option, respondents were asked whether their households had (i) pursued the livelihood and (ii) how much income they had derived from the livelihood for each year from 2015-2021.

Livelihood Categories	Specific Livelihoods
**Farming**	Rice and Paddy Plantation<br>Maize Plantation<br>Wheat Plantation<br>Mustard/Lentil Plantation<br>Fruit, Vegetable, Fodder, Tree and Other Farming
**Livestock**	Large animals (cattle and buffaloes)<br>Small animals (goat, sheep, pigs)<br>Chicken, ducks, and poultry farming
**Migration**	Household members working outside Chitwan Valley<br>Household members working outside Nepal
**Off-Farm Labor**	Own Business<br>Salary Job<br>Wage Labor
**Other**	Pension/Allowance/Interest/Bonus<br>Other

Table 4: List of Livelihood Categories

3 Descriptive Statistics

This section provides further information and visualization of descriptive statistics that are referenced in the main text, Section 3.1.

3.1 Exposure to Climate-Related Hazards

To investigate the effects of exposure to climate-related hazards in shaping livelihood diversification strategies, we asked households to self-report if they had experienced each of 7 hazards in each year from 2015-2021. Summary statistics of the proportion of households in our sample self-reporting exposure to each hazard in each year are shown in the main text. We find an increase over the study period in the proportion of households experiencing climate-linked shocks generally, and especially exposure to floods, whch increased from 11 percent of households in 2015 to 57 percent in 2021.

3.2 Perceptions of Livelihood Risks

To better understand how perceptions of farming risk compare to perceived risks of other livelihood alternatives, we asked respondents to rate the overall riskiness of each of 12 livelihood categories (SI Fig. 1b). In this context, farming is perceived as one of the most risky livelihood strategies available to Chitwan farmers, with approximately 61 percent of respondents identifying farming cereal crops as "High Risk". However, some of the most common alternatives, including international migration (78 percent) and off-farm wage labor (60 percent), were also perceived to be highly risky by the majority of respondents.

3.3 Household Income Sources

The average composition of household income by livelihood category for each year in our study period is displayed in the Main Text, Fig. 3a. Averaged across all study years, migration remittances and income from off-farm labor account for 31.6 and 33.6 percent of household income, respectively, with only 16.8 and 8.8 percent of income coming directly from livestock and farming (i.e., selling crops), respectively.[1] This likely reflects the predominance of subsistence agriculture in the Chitwan District; farming households generally consume most of what they grow, and there are limited commercial markets to sell crop harvests for income. While migration and off-farm labor both account for substantial proportions of farming households' income in aggregate, individual household income portfolios vary substantially (SI Fig. 2). Approximately half of all households receive no income from either remittances and/or off-farm labor, while approximately 20 percent of households receiving at least 80 percent of their income from one of these sources.

[1] Note that an average of 9.2 percent of household income comes from other livelihood activities.

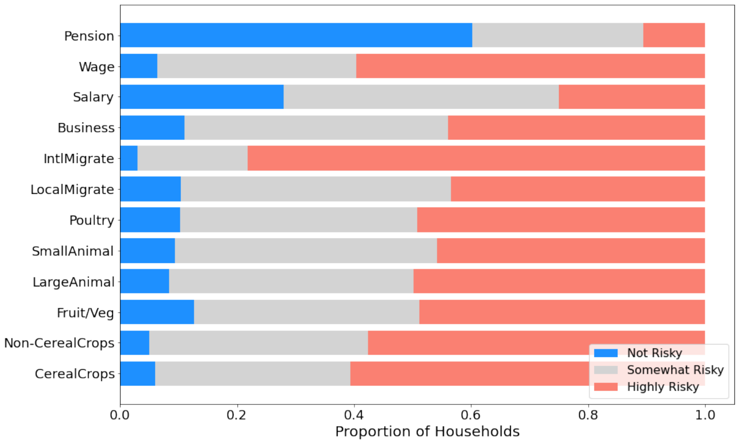

(a) **Livelihood Risk Perceptions**

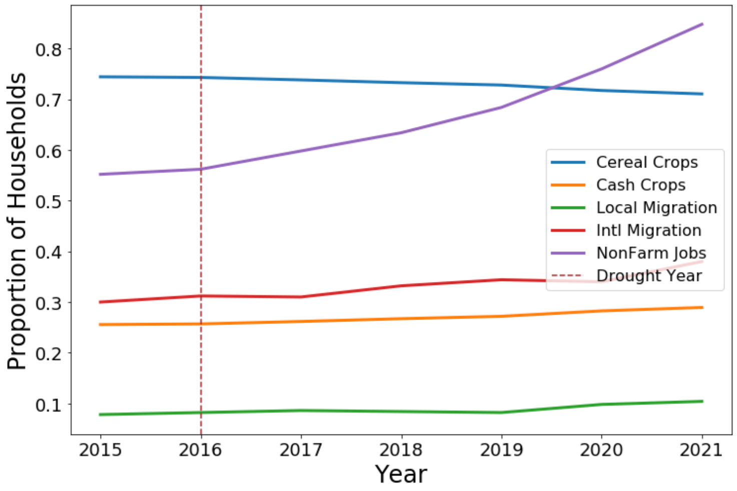

(b) **Household Livelihood Choices**

Figure 1: **a)** For each of 12 livelihood categories, the proportion of respondents assessing a livelihood to be "Not Risky" (blue), "Somewhat Risky" (gray), and "Highly Risky" (red) are shown by the horizontal bars. **b)** Distribution of survey sample households by self-reported livelihoods, 2015-2021.

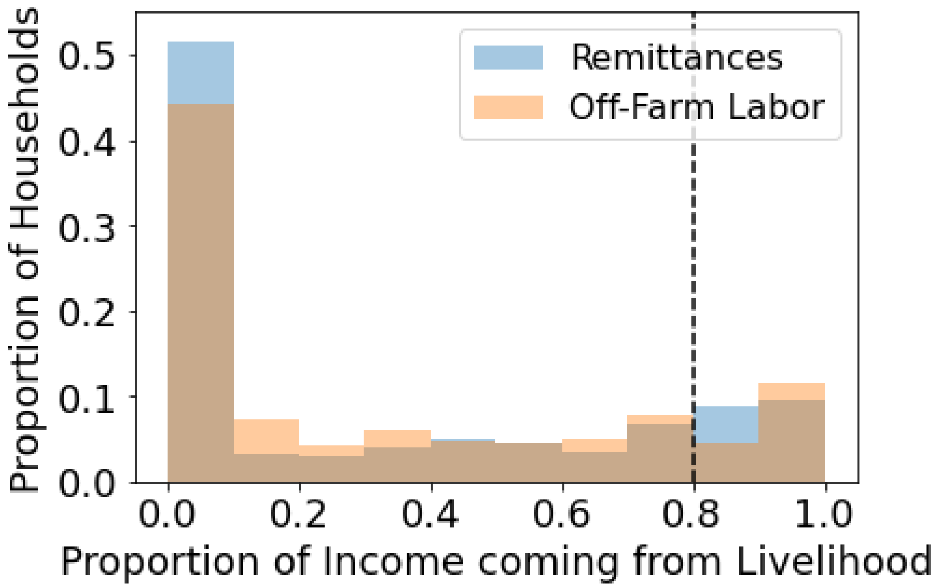

Figure 2: **Distribution of households by proportion of income coming from remittances (blue) and off-farm labor (orange)**. While each of these livelihoods account for a substantial proportion of aggregate income among farming households, approximately half of all households do not engage in at least one of these activities.

3.4 Geospatial Analysis of Key Variables

While the level of analysis in the main text is conducted at the individual household scale, a geospatial analysis of key variables may also reveal important insights about the relationship between information sources, climate risk perceptions, and income composition. In this section, variables are aggregated at the subward scale. There are six such subwards in our analysis (three each belonging to Ward 23, by the East Rapti River in the south, and Ward 26, by the Narayani River in the north), ranging from 49-106 respondents per subward.

Fig. SI 3a-d displays mean values of household income, climate risk index, and exposure to droughts and floods by subward (darker shadings indicate higher values for the reported variable). The highest mean climate risk perception occurs in Subward 26-10, along the banks of the Narayani River ($\tilde{R} = 0.54$), while the lowest mean perception occurs in the upland Subward 23-01 ($\tilde{R} = 0.32$). However, there is no discernible geographic pattern in the rank order of mean climate risk perceptions: the second and third highest mean perceptions occur in Subwards 26-05 (upland) and 23-04 (riverine), respectively. There also does not appear to be a direct relationship between locations with higher rates of self-reported drought/flood exposure and generalized risk perceptions. On the one hand, Subward 26-10 reports the highest rate of drought exposure (39.2 percent), second-highest rate of flood exposure (35.4 percent), and the highest generalized climate risk perception. On the other hand, Subward 26-11 reports the highest rate of flood exposure (38 percent) second-highest rate of drought exposure (36.5 percent), but the second-lowest generalized climate risk perception (0.34). There also does not appear to be an obvious connection to annual household income: while the poorest subward (26-10; 250,000 NRs/year) reports the highest mean risk perception, the next-poorest (23-01; 280,000 NRs/year) reports the lowest mean risk perception. The highest-income subward (23-04; 370,000 NRs/year) ranks third in generalized climate risk perception. This suggests that other factors may be influencing climate risk perceptions.

SI Figure 3e-h illustrates the mean proportion of household income coming from each of four livelihood categories: farming, livestock, off-farm labor, and migration remittances. There are some general trends to note in this regard: households in subward 26 (by the Narayani River) generally rely more on farming income, whereas farmers in subward 23 exhibit greater reliance on livestock income. Households in inland subwards (23-01 and 26-05) tend to rely more on migration remittance income, whereas households in riverine subwards exhibit greater reliance on off-farm labor income. This may lend credence to the theory that climate shocks, especially in the form of floods, pose an obstacle to diversifying income sources away from farming, especially through migration.

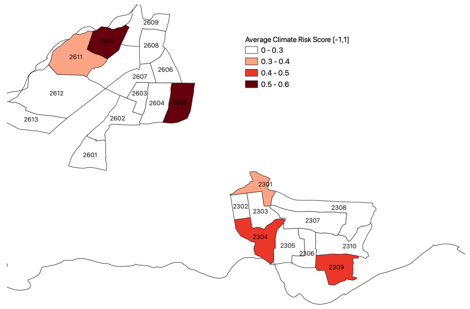

(a) **Climate Risk Index**

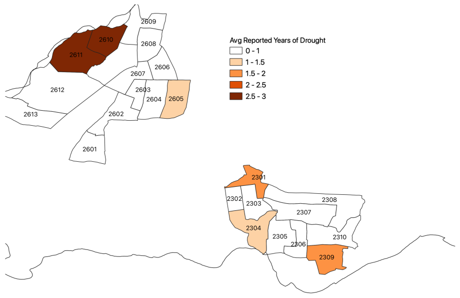

(b) **Drought Exposure**

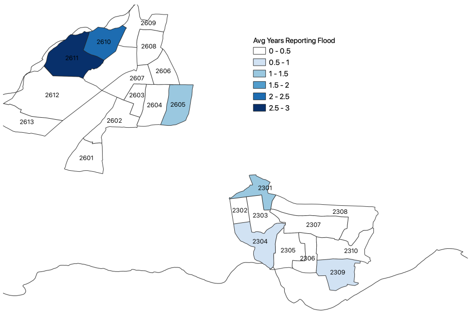

(c) **Flood Exposure**

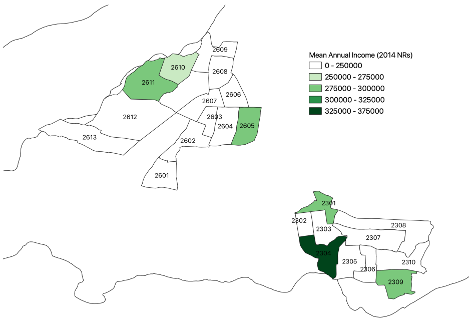

(d) **Household Income**

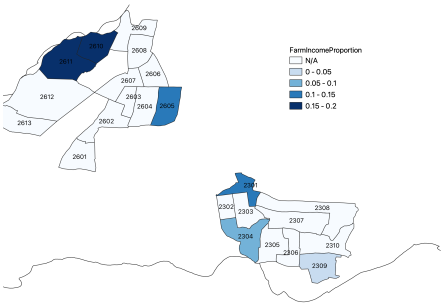

(e) **Farm Proportion**

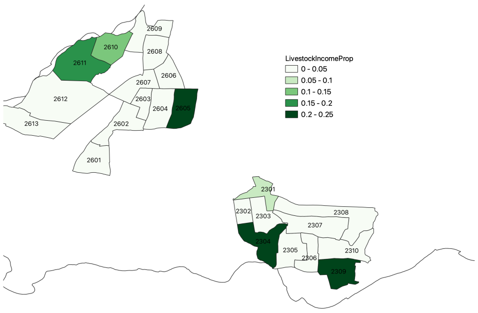

(f) **Livestock Proportion**

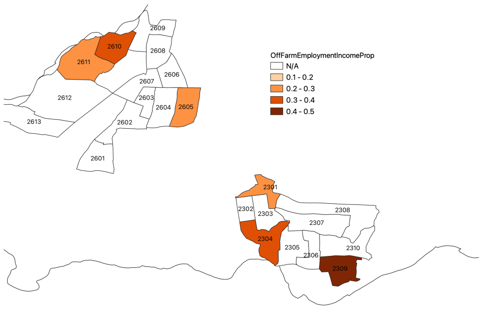

(g) **Off-Farm Labor Proportion**

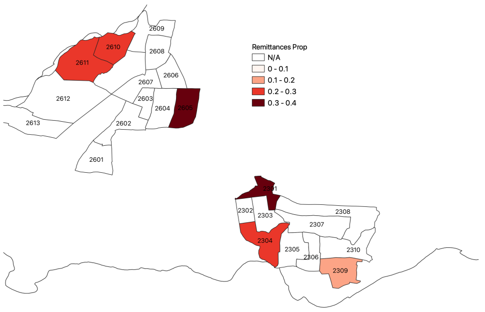

(h) **Remittance Proportion**

Figure 3: **Spatial distribution of climate exposures and income sources.** Maps display the distribution of **a)** the aggregate climate risk index, **b-c)** exposures to droughts and floods, **d)** mean household income, and **e-h)** proportion of income derived from various livelihood activities, averaged across our study period for each of the six sub-wards in the study area. In all panels, darker shadings represent higher values for the reported variable.

3.5 Hazard Comparison with Remotely-Sensed Data

In the Main Text and in SI Fig. 1, we summarize respondents' self-reported experience with climate-linked hazards from 2015-2021. While these responses generally match long-term climatic trends in the region [7, 11], we also compare these to remotely-sensed data from the Chitwan region over the same study period. Specifically, we use the Standardized Precipitation and Evapotranspiration Index (SPEI), a global database of soil moisture that is collected at monthly intervals for 0.5 x 0.5 degree grid cell (roughly 50 km by 50 km at the Equator). Positive values of the SPEI indicate that the soil moisture of a given region is higher than its historical average (starting from 1901) for the same time in the year, whereas negative values indicate a soil moisture deficit relative to this historical baseline. It is measured over multiple time increments ranging from 1 to 48 months; here, we use the three month increment (SPEI-03) as a common metric for measuring agricultural drought.

Our analysis of SPEI03 data indicates that while the Chitwan District experienced both extreme moisture and extreme drought during our study period of 2015-2021 (measured as 1 standard deviation above or below the historical baseline), these events actually decreased in frequency compared to the preceding years (Fig. SI 4). This runs counter to the perceptions of a majority of respondents to our survey, who generally perceive the risk of floods and droughts to be increasing over time (Main Text, 3.1). However, an important caveat is that the SPEI is a geographically coarse measure and does not capture variation in exposure to floods and droughts within the Chitwan District. For example, it may be that farmers near the district's two rivers did in fact experience greater flooding over the study period, even if the average soil moisture over the entire region remained within 1 standard deviation of the historical baseline. The discrepancy between trends in SPEI and trends in farmer perceptions could also reflect greater farmer awareness and sensitivity to climate-linked hazards, whereby even relatively smaller events are now perceived as notable.

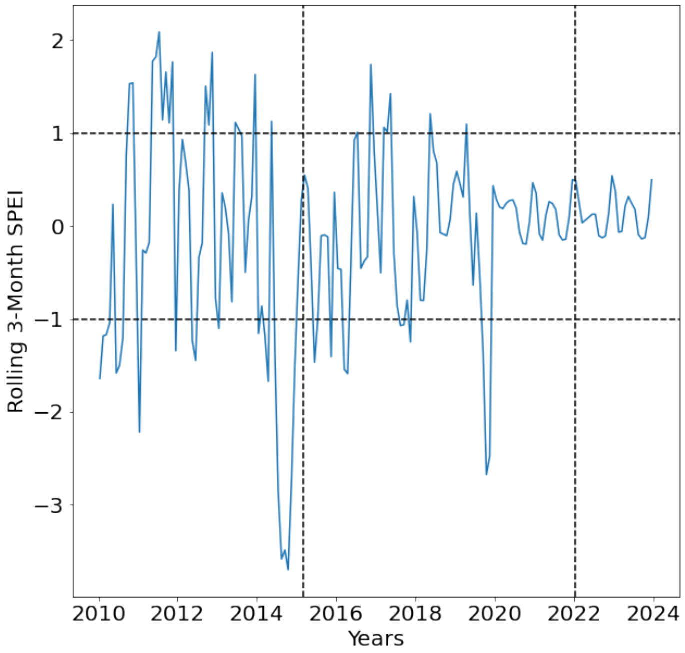

(a) **Chitwan District Standardized Precipitation and Evapotranspiration Index-03 Values, 2010-2023**

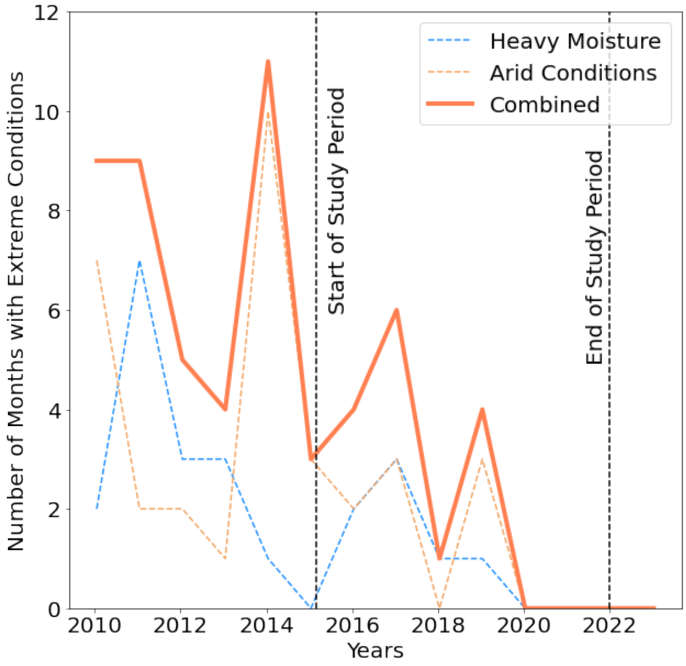

(b) **Months with Extreme Events, 2010-2023**

Figure 4: **Extreme Events in Chitwan District based on Standardized Precipitation and Evapotranspiration Index (SPEI) Measurements.** The Standardized Precipitation and Evapotranspiration Index is a database that measures the frequency of moisture-related extreme events. **a)** Here, we show the rolling 3-month SPEI value for the grid cell containing Bharatpur (the main municipality in Chitwan) from 2010-2023. Values above 1 indicate extremely wet conditions (1 standard deviation in soil moisture above historical average), whereas values below 1 indicate extremely dry conditions (1 standard deviation below historical average). Vertical dashed lines indicate the starting and ending year of the study period. **b)** We display the number of months each year in which the SPEI was above 1 (blue dotted line), below 1 (orange dotted line), and the combined months with extreme conditions (solid red line). There is a general downward trend in the frequency of extreme conditions during the study period.

4 Alternative Model Specifications

In this section, we explore several alternative specifications of key variables, econometric models, and their implications for the robustness of the results presented in the main text.

4.1 Effect of Specific Hazards on Perceptions of Livelihood Risk

In the Main Text, Section 3.2, we investigate how farmers generalized climate risk perceptions are related to their perceptions of different livelihood options. Here, we further develop a hazard-specific analysis on farmers' perceptions of livelihood risks to complement the analysis of generalized climate risk perceptions on farmer perceptions of overall livelihood riskiness. We present results in Table 5 here. Key results are referenced in Section 3.2 of the main text.

**Variable**	**Cereal Crops**	**Large Animals**	**Wage Labor**	**Intl Migration**	**Pension**
Female	0.0292	0.180	0.206	-0.341	0.326
	(0.225)	(0.217)	(0.221)	(0.287)	(0.220)
Age	-0.0002	-0.0094	-0.0101	-0.0017	-0.0025
	(0.009)	(0.009)	(0.009)	(0.011)	(0.009)
Secondary School	0.383*	0.0046	-0.227	0.535*	0.0071
	(0.227)	(0.219)	(0.229)	(0.284)	(0.222)
Household Size	0.0283	0.0108	0.0298	0.0637	0.0275
	(0.056)	(0.053)	(0.056)	(0.068)	(0.054)
Land Area Operated	0.0121	0.0130	0.0068	-0.0226**	-0.0048
	(0.009)	(0.009)	(0.009)	(0.010)	(0.010)
Near River	-0.137	-0.0398	-0.0270	0.705**	-0.239
	(0.242)	(0.229)	(0.240)	(0.281)	(0.232)
Drought Risk	0.271**	0.346***	0.0698	0.556***	0.178
	(0.127)	(0.125)	(0.128)	(0.152)	(0.130)
Flood Risk	0.0624	-0.129	0.116	-0.0025	0.304**
	(0.135)	(0.132)	(0.134)	(0.164)	(0.142)
Heat Risk	0.144	0.320***	0.291**	-0.183	-0.148
	(0.126)	(0.121)	(0.126)	(0.158)	(0.125)
Pest Risk	0.478**	0.285	0.337*	0.111	0.210
	(0.197)	(0.198)	(0.199)	(0.231)	(0.214)
Groundwater Risk	−0.0829	0.0997	0.0293	-0.192	0.0591
	(0.122)	(0.117)	(0.120)	(0.154)	(0.120)
Hail Risk	0.326**	0.378***	0.175	0.504***	0.0574
	(0.128)	(0.125)	(0.127)	(0.162)	(0.123)
Frost Risk	0.0167	0.237*	0.256*	0.429***	0.104
	(0.139)	(0.132)	(0.135)	(0.163)	(0.138)
Social Networks ($\tilde{G}$)	-0.173*	-0.227**	0.0379	-0.216*	0.0384
	(0.100)	(0.099)	(0.102)	(0.123)	(0.101)
Information Sources ($\tilde{I}$)	-0.145	-0.0759	-0.316***	-0.0537	-0.173*
	(0.098)	(0.096)	(0.097)	(0.117)	(0.101)

Table 5: **Specific Climatic Drivers of Livelihood Risk Perceptions.**

4.2 Principal Component Analysis of Risk Perceptions

As an additional alternative specification, we conduct a principal components analysis of latent factors that may be driving climate risk perceptions. Table 6 displays correlations between the six indicators used in our survey to measure risk perceptions of climate-driven hazards. As some of these indicators exhibit moderate levels of correlation (e.g., perceptions of frost and pests, and heat and frost exhibit correlations $\rho > 0.25$), one alternative specification of perceived climate risk is to decompose such variables into their principal components. That is, rather than weight all indicators equally in a single index that may be skewed due to correlations between the indicators, an alternative would be to specify the orthogonal latent factors that drive variation across these six indicators.

In Table 7, we illustrate the factor loadings with two such latent factors. The first latent factor is strongly associated with variance in perceptions of flood risk (96 percent of variance in flood risk is explained by latent factor 1), and has weaker associations with perceived risk of groundwater availability (16 percent of variance), drought (11 percent), and heat (11 percent). We call this factor a "Water Balance" risk factor, given its association with water-related hazards. The second latent factor is strongly associated with perceptions of frost risk (62 percent of explained variance), pests (52 percent), and heat (42 percent), and lesser associations with the other hazards. We call this factor a "Withering" risk factor for its association with hazards that can cause initially healthy crop plants to wither over time. Each latent factor explains approximately 16 percent of the total variance in household perceptions along the six hazards.

To assess whether this alternate specification of climate risk perception affects our main results, we replace the composite climate risk factor $\tilde{R}_i$ in the Main Text, Section 2.4, with these two latent factors, F_i and W_i. These results are shown in Table 8. Generally, we find consistent sizes and significance of effects for each explanatory variable as in the results shown in Section 3.2, Table 5. In general, neither of these risk perception factors are significantly associated with substantial changes in household income composition, although an increased Withering Factor perception score is weakly associated with an increase in reliance on livestock income (2.4 percentage points, $p < 0.1$). As in the main specification, farmers' risk perceptions of off-farm labor is significantly and negatively associated with reliance on this strategy for household income, while perception of farming risk is significantly and positively associated with higher reliance on this strategy for income. Another similar conclusion is that exposure to either floods or droughts in a given year is significantly and positively associated with reliance on farming; while exposure to drought is significantly and negatively associated with reliance on off-farm labor for income.

	Droughts	Floods	Groundwater	Heat	Pests	Frost
Droughts	1.0	0.18	-0.043	0.16	0.15	0.11
Floods	0.18	1.0	0.16	0.22	0.15	0.17
Groundwater	-0.043	0.16	1.0	0.0099	-0.020	0.053
Heat	0.16	0.22	0.0099	1.0	0.18	0.27
Pests	0.15	0.15	-0.020	0.18	1.0	0.33
Frost	0.11	0.17	0.053	0.27	0.33	1.0

Table 6: Pearson's Correlation Coefficients between Natural Hazard Risk Perceptions.

Factor	1 (Water Balance)	2 (Withering Factor)
Droughts	0.11	0.25
Floods	0.96	0.27
Groundwater	0.16	0.00
Heat	0.11	0.42
Pests	0.010	0.52
Frost	0.011	0.62
**Total Explained Variance**	**0.16**	**0.16**

Table 7: Factor Loadings for Natural Hazard Risk Perceptions.

Variable	Farming	Livestock	Off-Farm Labor	Remittance
Constant	0.901**	0.158	-0.354***	0.684
	(0.415)	(0.512)	(0.045)	(0.652)
Female	-0.0181***	0.0028	-0.107***	0.0850
	(0.003)	(0.088)	(0.002)	(0.093)
Age	-0.0040	0.0506***	-0.0758***	-0.0096**
	(0.003)	(0.010)	(0.009)	(0.004)
Secondary School	-0.0043	0.0167	-0.124***	0.0307
	(0.007)	(0.017)	(0.011)	(0.029)
Household Size	-0.0160***	-0.0070	-0.0012	0.0185***
	(0.001)	(0.011)	(0.004)	(0.006)
Land Area Operated	0.0706***	0.0189***	-0.0551***	-0.0356***
	(0.006)	(0.000)	(0.006)	(0.009)
Near River	-0.0379	0.0349	0.111***	-0.115***
	(0.052)	(0.078)	(0.006)	(0.008)
Water Balance	0.0010	-0.0059***	-0.0200***	0.0292*
	(0.005)	(0.001)	(0.006)	(0.017)
Withering Factor	0.0065	0.0240***	-0.0208***	-0.0088*
	(0.014)	(0.003)	(0.002)	(0.005)
Social Networks ($\tilde{G}$)	0.0117	0.0102	-0.0242	0.0061
	(0.018)	(0.020)	(0.018)	(0.024)
Information Sources ($\tilde{I}$)	0.0009	0.0099	-0.0247	0.0308
	(0.006)	(0.019)	(0.022)	(0.048)
Flood Exposure	-0.0201***	-0.0046	0.0041	-0.0217***
	(0.002)	(0.026)	(0.035)	(0.005)
Drought Exposure	0.0190	0.0102	-0.0636**	0.0032
	(0.014)	(0.025)	(0.030)	(0.026)
Year	-0.0089	0.0002	0.0100 ***	-0.0064
	(0.006)	(0.006)	(0.001)	(0.008)

Table 8: **Drivers of Changes to Annual Household Income Composition with Latent Climate Risk Perception Factors.**

4.3 Alternate Measures of Social Capital

In the Main Text, we construct the social group index $\tilde{G}_i$ through an aggregate count of all social groups in which respondent i participates (see SI 2.1 for more details on this calculation). However, other measures of social capital may also provide relevant information in this context. Here, we explore two such alternative measures: (i) disaggregating our social group index by membership in specific groups, and (ii) assessing capital by the number of close friends with whom a respondent would discuss economic, migration, and other livelihood decisions.

4.3.1 Disaggregating Social Groups

In this section, we disaggregate our index $\tilde{G}_i$ to analyze the degree to which membership in specific social groups may influence livelihood diversification. Specifically, it may be that farming-based social groups are (i) more relevant than others for disseminating information regarding climate risks to farming and potential adaptation options, and/or (ii) may exhibit specific social norms that would dissuade group members from reducing their investments in farming. Here, we construct an alternative version of the index, which we call the "Farming Group Index" ($\bar{G}_i^F$), as a way to measure the effects of the two farming-specific social groups in our survey: farming cooperatives and livestock groups. We construct this index in the same manner as described in SI 2.1, but only count membership in these two farming-based groups in this alternative specification.

Drivers of households' annual income composition using the farming group index are displayed in SI Table 9. Here, there are a few important differences when compared to the main model presented in the Main Text, Section 3.2. Notably, whereas our more generalized measure of social group membership exerts significant effects on the proportion of income coming from farming and off-farm livelihoods, membership in farming-specific social groups is not significantly associated with changes to income composition. This is somewhat counterintuitive, as our expectation would be that farming-specific groups would drive the positive effect of social networks on farming income that we found in the main text. This discrepancy may indicate that other social groups - including women's groups, youth groups, community forest groups, and lending groups - are also important indicators of the richness of a household's social network, which in turn may embed households even further in local farming-based livelihoods.

4.3.2 Measuring Social Capital by Number of Friends

An alternative to measuring social capital by membership in groups is to consider the number of individuals with whom an individual may share information and/or material resources. In our survey, we asked respondents to specify the number of close friends, relatives, and neighbors with whom they regularly spoke about farming, migration, or other livelihood strategies. Respondents were presented with five-person increments (i.e., "None", "1-5", "6-10", "11-15", etc.) and were asked to select one such increment. A majority of respondents selected the "1-5" increment, and 75 percent of respondents selected either "1-5" or "6-10".

Here, we estimate our two main regression specifications using the number of friends as the social capital indicator, rather than number of social groups. For the purposes of this analysis, we estimate each individual respondent's number of friends as the midpoint of each 5-person increment (i.e., 2.5, 7.5, 12.5, etc.). Generally, our results for factors driving income diversification (Table 10) do not substantially diverge from results in the Main Text using our social group index (Section 3.2). One exception is the role of social capital itself; as measured by the number of friends, social capital appears to be positively correlated with the proportion of income derived from farming and livestock, and negatively correlated with the proportion coming from off-farm employment. In the main specification, social

capital was not significantly correlated with how respondents derived their income. While this discrepancy provides some nuance regarding the role of social capital in shaping income portfolios, it does not substantially affect our findings regarding the influence of droughts, floods, and climate risk perceptions on income diversification.

**Variable**	**Farming**	**Livestock**	**Off-Farm Labor**	**Remittance**
Constant	0.897**	0.149	-0.359***	0.705
	(0.400)	(0.507	(0.038)	(0.667)
Female	-0.0157***	0.0051	-0.112***	0.0843
	(0.001)	(0.088)	(0.003)	(0.088)
Age	-0.0023	0.0526***	-0.0779***	-0.0116**
	(0.006)	(0.011)	(0.010)	(0.005)
Secondary School	0.0002	0.0208	-0.133***	0.0297
	(0.013)	(0.016)	(0.014)	(0.033)
Household Size	-0.0154***	-0.0067	-0.0022	0.0189***
	(0.001)	(0.008)	(0.005)	(0.003)
Land Area Operated	0.0721***	0.0190***	-0.0588***	-0.0340***
	(0.008)	(0.000)	(0.007)	(0.010)
Near River	-0.0343	0.0370	0.101***	-0.106***
	(0.059)	(0.088)	(0.012)	(0.030)
Composite Risk($\tilde{R}_i$)	0.0028	0.0127	-0.0215**	0.0069
	(0.011)	(0.009)	(0.008)	(0.008)
Farming Groups ($\tilde{G}_i^F$)	0.0036	0.0127	-0.0044	-0.0032
	(0.011)	(0.029)	(0.009)	(0.016)
Source Index ($\tilde{S}_i$)	0.0026	0.0111	-0.0284	0.0310
	(0.009)	(0.020)	(0.020)	(0.048)
Flood Exposure	0.0211***	-0.0050	-0.0016	-0.0152***
	(0.003)	(0.028)	(0.035)	(0.002)
Drought Exposure	0.0182	0.0115	-0.0593**	-0.0029
	(0.014)	(0.026)	(0.029)	(0.023)
Year	-0.0090	0.0002	0.0104 ***	-0.0068
	(0.006)	(0.006)	(0.001)	(0.008)

Table 9: **Drivers of Changes to Annual Household Income Composition with Farm-Specific Social Groups.**

**Variable**	**Farming**	**Livestock**	**Off-Farm Labor**	**Remittance**
Constant	0.885**	0.136	-0.326***	0.699
	(0.388)	(0.525)	(0.005)	(0.658)
Female	-0.0151***	0.0073	-0.113***	0.0836
	(0.001)	(0.080)	(0.002)	(0.086)
Age	-0.0028	0.053***	-0.0762***	-0.0122***
	(0.006)	(0.007)	(0.008)	(0.004)
Secondary School	-0.00001	0.0231	-0.131***	0.0280
	(0.014)	(0.030)	(0.011)	(0.037)
Household Size	-0.0154***	-0.0068	-0.0023	0.0190***
	(0.001)	(0.009)	(0.005)	(0.003)
Land Area Operated	0.0731***	0.0213***	-0.0610***	-0.0341***
	(0.009)	(0.004)	(0.008)	(0.013)
Near River	-0.0369	0.0322	0.107***	-0.106***
	(0.056)	(0.081)	(0.010)	(0.025)
Composite Climate Risk ($\tilde{R}$)	0.0021	0.0129**	-0.0190***	0.0060
	(0.011)	(0.005)	(0.007)	(0.007)
Number of Friends	0.0027***	0.0029*	-0.0075**	0.0014
	(0.001)	(0.002)	(0.003)	(0.001)
Information Sources ($\tilde{I}$)	0.0009	0.0106	-0.0232	0.0294
	(0.010)	(0.015)	(0.021)	(0.047)
**Flood Exposure**	**0.0204*****	**-0.0060**	**0.0004**	**-0.0155*****
	**(0.004)**	**(0.030)**	**(0.039)**	**(0.001)**
**Drought Exposure**	**0.0185**	**0.0121**	**-0.060****	**-0.0029**
	**(0.014)**	**(0.025)**	**(0.027)**	**(0.023)**
Year	-0.0089	0.0002	0.0103***	-0.0068
	(0.006)	(0.007)	(0.000)	(0.008)

Table 10: **Factors affecting household income composition, with social capital measured as number of friends**.

4.4 Decomposition of Effects by Income Quartiles

Tables 11 - 14 present results of the decomposition of the effects of demographic factors, social capital, and hazard exposure on household income composition. These results are used to generate Figure 6 in the Main Text, Section 3.2, and Fig. 5 in this section.

DV = Farm Income Proportion	1 (lowest)	2	3	4 (highest)
Constant	1.48*	0.439	0.622	0.917***
	(0.834)	(0.346)	(0.414)	(0.01)
Female	-0.0472	0.0331	-0.0058	-0.0283***
	(0.029)	(0.025)	(0.009)	(0.002)
Age	0.0031	0.0011	-0.0104	-0.0069**
	(0.005)	(0.012)	(0.018)	(0.003)
Secondary School	0.0555***	0.0296	-0.0214	0.0030
	(0.003)	(0.048)	(0.037)	(0.045)
Household Size	-0.0270***	-0.0054	-0.0097**	-0.0036
	(0.003)	(0.012)	(0.004)	(0.003)
Land Area Operated	0.102***	0.0924***	0.0702***	0.0192**
	(0.038)	(0.016)	(0.002)	(0.010)
Near River	-0.116	-0.0488	-0.0158	0.0012
	(0.076)	(0.039)	(0.032)	(0.010)
Composite Risk($\tilde{R}$)	-0.0023	0.0151	-0.0008	0.0069***
	(0.020)	(0.013)	(0.008)	(0.00006)
Social Networks ($\tilde{G}$)	0.061	0.0365*	-0.0025	0.0009
	(0.063)	(0.019)	(0.015)	(0.006)
Information Source ($\tilde{I}$)	0.0124***	-0.0028	0.0063	0.0132
	(0.003)	(0.011)	(0.017)	(0.027)
Flood Exposure	0.0506***	-0.0144***	-0.0018	0.0391***
	(0.016)	(0.005)	(0.007)	(0.008)
Drought Exposure	-0.0088	0.0254	0.0194	0.0294
	(0.027	(0.025)	(0.022)	(0.034)
Year	-0.0139	-0.0037	-0.0061	-0.011***
	(0.011)	(0.006)	(0.005)	(0.00)

Table 11: **Drivers of Changes to Annual Household Farming Income Composition**

**DV = Livestock Income Proportion**	**1 (lowest)**	**2**	**3**	**4 (highest)**
Constant	0.0219	-0.406*	0.624	0.470
	(0.418)	(0.219)	(0.492)	(0.588)
Female	0.0194	0.0455	-0.0346	-0.0026
	(0.067)	(0.077)	(0.130)	(0.074)
Age	0.0588***	0.0681***	0.0230	0.0441***
	(0.007)	(0.019)	(0.037)	(0.00003)
Secondary School	0.0344***	0.0061	0.0434	0.047***
	(0.011)	(0.0063)	(0.058)	(0.011)
Household Size	-0.0001	0.0074	-0.0132*	-0.0069
	(0.010)	(0.013)	(0.008)	(0.025)
Land Area Operated	0.0323	0.0127***	0.0362**	0.0208**
	(0.029)	(0.001)	(0.016)	(0.008)
Near River	-0.0012	0.0386	-0.0028	0.0657**
	(0.134)	(0.119)	(0.034)	(0.028)
Composite Risk($\tilde{R}$)	0.0256***	0.0070	0.0147	0.0158**
	(0.005)	(0.020)	(0.020)	(0.006)
Social Networks ($\tilde{G}$)	0.0164	0.0253	0.0379	-0.0079
	(0.017)	(0.041)	(0.045)	(0.005)
Information Source ($\tilde{I}$)	0.0036	0.0073	0.0200	0.025***
	(0.010)	(0.054)	(0.037)	(0.004)
Flood Exposure	-0.0175	-0.0564***	0.0827	-0.0214
	(0.015)	(0.011)	(0.060)	(0.021)
Drought Exposure	0.0424	0.0176	-0.0067	-0.079
	(0.065)	(0.037)	(0.032)	(0.062)
Year	0.0023	0.0067**	-0.0054	-0.0050
	(0.006)	(0.003)	(0.005)	(0.005)

Table 12: **Drivers of Changes to Annual Household Livestock Income Composition**

DV = Off-Farm Labor	1 (lowest)	2	3	4 (highest)
Constant	-0.498**	0.308	-1.176***	-0.217***
	(0.232)	(0.368)	(0.143)	(0.028)
Female	-0.124***	-0.219*	-0.0112	0.0192***
	(0.039)	(0.114)	(0.056)	(0.007)
Age	-0.127***	-0.112***	0.0086	-0.0109
	(0.022)	(0.024)	(0.029)	(0.034)
Secondary School	-0.203***	-0.171*	0.0164	-0.0555***
	(0.070)	(0.093)	(0.088)	(0.010)
Household Size	0.0049	-0.0266***	0.0062***	0.0194
	(0.012)	(0.008)	(0.001)	(0.025)
Land Area Operated	-0.114**	-0.0274***	-0.0812***	-0.0314*
	(0.046)	(0.003)	(0.021)	(0.018)
Near River	0.262***	0.0083	0.138	0.0009
	(0.007)	(0.104)	(0.107)	(0.005)
Composite Risk($\tilde{R}$)	-0.0423***	-0.0488**	0.0004	-0.0249
	(0.012)	(0.019)	(0.024)	(0.019)
Social Networks ($\tilde{G}$)	-0.0772**	-0.0951**	0.0203	0.0449
	(0.030)	(0.037)	(0.047)	(0.043)
Information Source ($\tilde{I}$)	-0.0187	-0.0350***	-0.0640***	-0.0208***
	(0.059)	(0.013)	(0.002)	(0.008)
Flood Exposure	0.0176	0.0792	-0.0747**	-0.0174***
	(0.011)	(0.049)	(0.030)	(0.005)
Drought Exposure	-0.112	-0.0524	-0.0866	0.0530
	(0.086)	(0.069)	(0.059)	(0.034)
Year	0.0103***	0.0050***	0.0181***	0.0057***
	(0.003)	(0.001)	(0.00008)	(0.001)

Table 13: **Drivers of Changes to Annual Household Off-Farm Labor Income Composition**

DV = Remittances	1 (lowest)	2	3	4 (highest)
Constant	0.324	1.53***	0.846*	0.348
	(0.213)	(0.504)	(0.656)	(1.30)
Female	0.0591	0.108	0.0210	0.0146
	(0.042)	(0.174)	(0.039)	(0.155)
Age	0.0051***	0.0017	-0.0328***	-0.0639
	(0.001)	(0.021)	(0.003)	(0.050)
Secondary School	0.0074	0.0332	-0.134***	-0.0406 **
	(0.058)	(0.053)	(0.023)	(0.018)
Household Size	0.0318***	0.0008	0.0016	-0.0055***
	(0.005)	(0.015)	(0.012)	(0.000)
Land Area Operated	-0.0203	-0.0640***	-0.0031	-0.0559*
	(0.019)	(0.007)	(0.009)	(0.033)
Near River	-0.161***	-0.0190	-0.186*	0.0406***
	(0.015)	(0.035)	(0.101)	(0.005)
Composite Risk($\tilde{R}$)	0.0111***	0.0166	-0.0002	0.0186***
	(0.003)	(0.034)	(0.017)	(0.004)
Social Networks ($\tilde{G}$)	0.0045	0.0267	-0.0465***	-0.0413
	(0.017)	(0.036)	(0.017)	(0.044)
Information Source ($\tilde{I}$)	0.0333	0.0171	0.0861	-0.0064
	(0.023)	(0.068)	(0.060)	(0.051)
Flood Exposure	-0.0174***	-0.0244	-0.0227**	0.0225
	(0.003)	(0.028)	(0.009)	(0.016)
Drought Exposure	0.0183	0.0460***	-0.0067	-0.0107
	(0.045)	(0.010)	(0.088)	(0.111)
Year	-0.0035	-0.0184***	-0.0040	0.0013
	(0.003)	(0.003)	(0.009)	(0.016)

Table 14: **Drivers of Changes to Annual Household Remittance Income Composition**

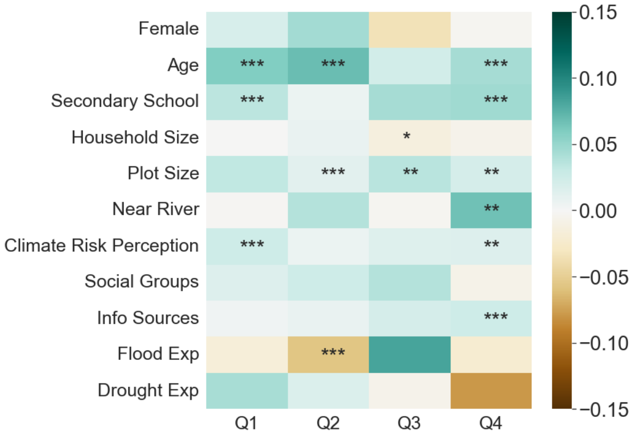

(a) **Livestock Proportion**

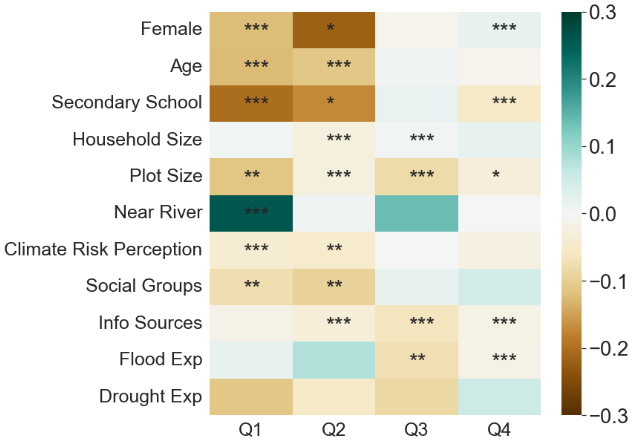

(b) **Off-Farm Labor Proportion**

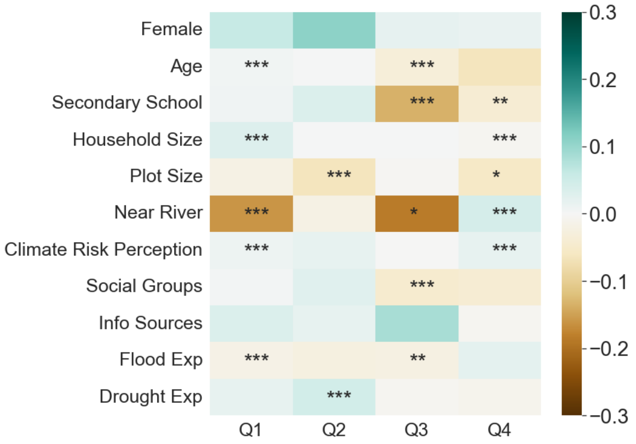

(c) **Remittances Proportion**

Figure 5: Effect Sizes and Significance on Income Proportion by Quartiles.

4.5 Endogeneity Tests

In the main text, we refer to several potential sources of endogeniety in our conceptual framework, in which our dependent variable (the degree of livelihood diversification away from farming) may exert a causal effect on our independent and/or intervening variables (Main Text, Fig. 1). If significant, such effects would lead to an erroneous specification of the effects we find in our main regression analyses in Section 3 of the main text. While we cannot fully investigate the direction of causality in the absence of a panel dataset constructed through multiple waves of data collection, here we present some initial tests to gain insight into these relationships. In particular, we test for four potential sources of endogeneity:

- $\hat{H}_1$: The degree of livelihood diversification away from farming affects perceived climate risks. For example, an increased reliance on farming for income may cause a household to be more attuned to the ways in which climate-driven risks affect their household income, and increase households' perceptions of climate risks.
- $\hat{H}_2$: The degree of livelihood diversification away from farming affects perceived riskiness of specific livelihoods, including both farm and non-farm livelihoods. For example, factors that lead to an increased reliance on farming for income may in turn cause respondents to view this livelihood as riskier. Alternatively, greater reliance on a given livelihood may provide a household with more experience and familiarity with that option, and reduce its perceived riskiness.
- $\hat{H}_3$ The degree of livelihood diversification away from farming affects households' diversity of information sources. For example, engaging in migration or off-farm labor may lead a household to seek out new information sources related to those livelihoods.
- $\hat{H}_4$: The degree of livelihood diversification away from farming affects households' average size of social networks. For example, engaging in a non-farm livelihood (e.g., migration) may increase a household's connections to new social groups (e.g., migrant networks). Alternatively, increased reliance on farming may lead households to seek out farming and other local social groups.

To test these hypotheses, we conduct regression analyses in which the originally-hypothesized dependent variable (the degree of diversification from farming) now serves an independent variable, and the potentially endogenous variable (i.e., perceived climate risk; perceived livelihood risk; information source index; social groups index) now serves as the dependent variable. If the diversification from farming exerts a significant effect on the potentially endogenous variable, this would represent a strong signal of the potential for reverse causality. Specifically, we construct the following model to test $\hat{H}_1$:

$$\tilde{R}_i = \beta_0 + \vec{\beta}_{1-6} \cdot \vec{X}_i + \beta_7 \cdot \bar{Y}_i^k + \beta_{8-9} \cdot \tilde{S}_i + \epsilon_i \quad (6)$$

Which is a modification of Equation 3 in Section 2.4 of the main text. The differences in this model are that the dependent variable is now the perceived climate risk of household i, $\tilde{R}_i$, and the addition of independent variable $\bar{Y}_i^k$, which represents the average proportion of household i's income coming from livelihood k during the study period (2015-2021). This test does not display strong evidence of endogeneity between a household's income source and the respondent's climate risk perception (Table 15). The one possible exception is livestock income, which exerts a small effect at the $p < 0.10$ significance level on perceived climate risks. In our main results, we find that a household's general perceived climate risk is a significant factor in their perceptions of several livelihood risks (Main Text, Section 3.2). In terms of income dependence, higher perceived climate risks are also associated with higher dependence on livestock income, and decreased reliance on off-farm employment income (Main Text, Section 3.3). One hypothesis for these results is that livestock may serve as a "first-choice" income diversification strategy for households that perceive climate risks

to farming, but whom are less comfortable or familiar with non-farm livelihoods (e.g., off-farm employment and migration). This may push households perceiving high climate risks to invest more in livestock; in turn, this new investment may lead some households to still worry about how climatic risks affect the health and productivity of their livestock. While the direction of causality for this relationship is difficult to ascertain without robust panel data, we note that there is stronger statistical evidence for climate risks shaping dependence on livestock income than the reverse.

**DV = Climate Risk Perception ($\tilde{R}_i$)**	**Farming**	**Livestock**	**Wage Labor**	**Remittances**
Constant	-0.0120	-0.0360	0.0795	-0.0118
	(0.156)	(0.154)	(0.161)	(0.154)
Female	-0.0661	-0.0697	-0.0886	-0.0729
	(0.103)	(0.103)	(0.104)	(0.104)
Age	0.0721	0.0560	0.0568	0.0725
	(0.053)	(0.054)	(0.054)	(0.053)
Secondary School	0.0355	0.0297	0.0116	0.0335
	(0.112)	(0.112)	(0.115)	(0.113)
Household Size	0.0891**	0.0897**	0.0857*	0.0841*
	(0.045)	(0.045)	(0.045)	(0.045)
Land Area Operated	-0.0203	-0.0481	-0.0539	-0.0403
	(0.019)	(0.053)	(0.053)	(0.053)
Near River	-0.0503	0.0148	0.0443	0.0300
	(0.055)	(0.109)	(0.109)	(0.110)
Source Index ($\bar{S}_i$)	-0.0566	-0.0589	-0.0612	-0.0580
	(0.048)	(0.048)	(0.049)	(0.048)
Group Index ($\bar{G}_i$)	0.0237	0.0216	0.0205	0.0248
	(0.049)	(0.049)	(0.049)	(0.049)
**Income Proportion**	**0.107**	**0.294***	**-0.195**	**0.0620**
	**(0.254)**	**(0.175)**	**(0.131)**	**(0.142)**

Table 15: **Factors Influencing Generalized Climate Risk Perceptions ($\tilde{R}_i$).** This series of regressions modifies those presented in Main Text, Section 2.4, by adding the mean proportion of household income deriving from the indicated livelihood (farming, livestock, migration remittances, and wage labor) as an independent variable. All models have the general climate risk perception index ($\tilde{R}_i$) as the dependent variable, and differ based on which livelihood income source is included as the bolded independent variable. Significance levels: $^*p < 0.1$, $^{**}p < 0.05$, $^{***}p < 0.01$.

To test hypothesis $\hat{H}_2$, we modify the ordered logistic regression in Equation 2 in Section 2.4 of the Main Text as follows:

$$Prob(P_i^k \geq j) = \frac{1}{1 + exp(-\alpha_j - \beta_{1-6} \cdot \vec{X}_i + \beta_7 * \bar{Y}_i^k + \beta_{8-9} * \tilde{S}_i)} \quad (7)$$

in which $\bar{Y}_i^k$ represents the average proportion of household i's income that derives from livelihood k over the study period, as above, and $Prob(P_i \geq j)$ represents the probability that respondent i rates the riskiness of livelihood k above level j. In this analysis, we find stronger endogeneity between the likelihood of rating international migration as risky and a household's dependence on remittances for income. Specifically, an increase of 1 percentage point in the average proportion of household income deriving from remittances decreases a respondent's likelihood of rating migration as risky by an odds ratio of 0.41:1 ($p < 0.01$). In other words, households exhibiting greater reliance on remittances are significantly more likely to believe that migration is less risky than households exhibiting lower reliance on this form of income.

Regarding possible sources of endogeneity, this analysis thus demonstrates stronger support for $\hat{H}_2$ than $\hat{H}_1$. For this reason, in our analysis of factors affecting livelihood diversification (Equations 3 and 4 of Section 2.4), we only include climate risk perceptions as an independent variable, and not households' perceptions of specific livelihood risks.

To test for hypotheses $\hat{H}_3$ and $\hat{H}_4$, we again modify Equation 3 in Section 2.4 to conduct an OLS regression with income sources as the independent variable, and the information source index ($\tilde{I}_i$) and social group index ($\tilde{G}_i$), respectively, as dependent variables:

$$\tilde{Y}_i = \beta_0 + \vec{\beta}_{1-6} \cdot \vec{X}_i + \beta_7 \cdot \bar{Y}_i^k + \beta_8 \cdot \tilde{S}_i + \epsilon_i \tag{8}$$

This is the same structure as SI Equation 6, except here we remove the social group index as a covariate when $\tilde{Y}_i = \tilde{I}_i$, and remove the information source index when $\tilde{Y}_i = \tilde{G}_i$, as these are each hypothesized dependent variables in this reverse causality check. As with $\hat{H}_1$, we test four models for both $\hat{H}_3$ and $\hat{H}_4$, with each model featuring one livelihood income source (farming, livestock, off-farm employment, and remittances) as the independent variable $\bar{Y}_i^k$. We do not find strong support for $\hat{H}_3$, the hypothesis that income diversification affects general access to information sources (Table 17). Our results demonstrate that off-farm employment is weakly correlated with a decrease in information sources, and an increase in reliance on migration remittances is weakly associated with a slight increase in information sources ($p < 0.1$ for both relationships), with small effect sizes (e.g., a 10 percentage point increase in household income from off-farm employment is associated with a 0.025 standard deviation decrease in the information source index, approximately equal to 0.05 information sources). Further, neither the proportion of income deriving from farming nor that from livestock is significantly associated with a change in information sources accessed. This provides some grounding to reject $\hat{H}_3$ as a major source of endogeneity.

We also do not find statistical support for $\hat{H}_4$, the hypothesis that livelihood diversification affects membership in social groups (Table 18). Rather, demographic and geographic variables (e.g., household size, the size of land area operated, and proximity to the one of the Chitwan District's two rivers) are all strongly and positively associated with higher membership in social groups. In the main text, we also find that membership in social groups is not positively associated with income sources (Main Text, Tables 5-6), though they do play a moderate role in reducing risk perceptions of various livelihoods (Main Text, Table 4). These results suggest that social groups in the Chitwan District are likely to be relatively stable and are neither affected by, nor substantially shape, short-term income diversification strategies. However, we re-iterate the finding from SI 4.3 that if social capital is measured by the number of close friends, it is positively associated with an increased reliance on farming income and decreased reliance on off-farm income.

Variable DV = Social Group Index	Cereal Crops	Large Animal	Wage Labor	Intl Migratio
Female	-0.0188	0.189	-0.212	-0.286
	(0.220)	(0.211)	(0.220)	(0.281)
Age	0.0009	-0.0072	-0.0083	-0.0042
	(0.009)	(0.009)	(0.009)	(0.011)
Secondary School	0.378*	0.0512	-0.146	0.408
	(0.223)	(0.214)	(0.227)	(0.276)
Household Size	0.0321	0.0127	0.0220	0.0736
	(0.056)	(0.053)	(0.055)	(0.069)
Land Area Operated	0.0079	0.0118	0.0071	-0.0231**
	(0.010)	(0.009)	(0.009)	(0.010)
Near River	-0.123	-0.0032	-0.0272	0.573**
	(0.239)	(0.225)	(0.238)	(0.275)
Climate Risk	0.159***	0.202***	0.177***	0.228***
	(0.037)	(0.036)	(0.037)	(0.045)
Information Sources	-0.127	-0.0672	-0.292***	-0.0509
	(0.096)	(0.094)	(0.095)	(0.114)
Social Networks	-0.193**	−0.204**	0.0396	-0.193
	(0.098)	(0.096)	(0.100)	(0.120)
**Livelihood**	**0.525**	**-0.275**	**-0.0292**	**-0.878***
**Income Proportion**	**(0.541)**	**(0.402)**	**(0.273)**	**(0.336)**

Table 16: **Factors affecting Perceived Riskiness of Different Livelihood Options**. This series of regressions modifies those presented in Main Text, 4.2, by adding the mean proportion of household income deriving from the indicated livelihood (farming, livestock, migration remittances, and wage labor) as an independent variable. Significance levels: $^{*}p < 0.1$, $^{**}p < 0.05$, $^{***}p < 0.01$.

**Variable (DV = Info Source Index)**	**Farming**	**Livestock**	**Wage Labor**	**Remittances**
Constant	0.105	0.0944	0.198	0.0503
	(0.155)	(0.150)	(0.157)	(0.157)
Female	-0.160	-0.162	-0.183	-0.180
	(0.111)	(0.111)	(0.112)	(0.111)
Age	-0.0960	-0.106*	-0.111*	-0.0927
	(0.061)	(0.063)	(0.061)	(0.061)
Secondary School	0.151	0.158	0.125	0.143
	(0.105)	(0.106)	(0.108)	(0.106)
Household Size	0.0717	0.0714	0.0687	0.0606
	(0.053)	(0.052)	(0.052)	(0.053)
Land Area Operated	0.108**	0.111**	0.102**	0.122**
	(0.054)	(0.049)	(0.049)	(0.049)
Near River	-0.118	-0.127	-0.0988	-0.0947
	(0.111)	(0.110)	(0.111)	(0.113)
Climate Risk	-0.0515	-0.0538	-0.0557	-0.0525
	(0.044)	(0.045)	(0.045)	(0.044)
Social Networks	0.184**	0.183***	0.180***	0.183***
	(0.045)	(0.046)	(0.045)	(0.045)
**Income Proportion**	**0.0915**	**0.179**	**-0.207***	**0.245***
	**(0.226)**	**(0.184)**	**(0.120)**	**(0.137)**

Table 17: **Reverse Causality Check for Information Source Index ($\tilde{I}_i$).** This series of regressions modifies those presented in Main Text, 4.1, by adding the mean proportion of household income deriving from the indicated livelihood (farming, livestock, migration remittances, and wage labor) as an independent variable. All models have the information source index ($\tilde{I}_i$) as the dependent variable, and differ based on which livelihood income source is included as the bolded independent variable. $\tilde{I}_i$ is a standardized index measuring the number of information sources accessed by a household at least once a year, in which a value of 1 is approximately equal to 2.17 information sources. Significance levels: $^{*}p < 0.1$, $^{**}p < 0.05$, $^{***}p < 0.01$.

**Variable (DV = Social Group Index)**	**Farming**	**Livestock**	**Wage Labor**	**Remittances**
Constant	-0.612***	-0.587***	-0.497***	-0.568***
	(0.140)	(0.140)	(0.1411)	(0.139)
Female	0.236**	0.229**	0.213**	0.229**
	(0.101)	(0.102)	(0.102)	(0.102)
Age	0.137***	0.126**	0.124**	0.137***
	(0.051)	(0.052)	(0.052)	(0.051)
Secondary School	0.403***	0.399 ***	0.382***	0.403***
	(0.099)	(0.099)	(0.101)	(0.100)
Household Size	0.0975**	0.0912**	0.0886**	0.0882**
	(0.045)	(0.045)	(0.045)	(0.045)
Land Area Operated	0.149***	0.168***	0.162***	0.173***
	(0.051)	(0.046)	(0.046)	(0.046)
Near River	0.269**	0.252**	0.275***	0.261**
	(0.105)	(0.105)	(0.106)	(0.106)
Climate Risk	0.0206	0.0189	0.0179	0.0216
	(0.043)	(0.043)	(0.043)	(0.043)
Info Sources	0.175***	0.175***	0.172***	0.176***
	(0.045)	(0.045)	(0.045)	(0.045)
**Income Proportion**	**0.317**	**0.188**	**-0.165**	**0.0222**
	**(0.253)**	**(0.197)**	**(0.117)**	**(0.122)**

Table 18: **Reverse Causality Check for Social Group Index ($\tilde{G}_i$).** This series of regressions modifies those presented in Main Text, 4.1, by adding the mean proportion of household income deriving from the indicated livelihood (farming, livestock, migration remittances, and wage labor) as an independent variable. All models have the social group index ($\tilde{G}_i$) as the dependent variable, and differ based on which livelihood income source is included as the bolded independent variable. $\tilde{G}_i$ is a standardized index measuring the number of social groups in which a household participates at least once a year, in which a value of 1 is approximately equal to 1.05 groups. Significance levels: $^{*}p < 0.1$, $^{**}p < 0.05$, $^{***}p < 0.01$.

References

[1] Ajzen, I. (1991). The theory of planned behavior. *Organizational Behavior and Human Decision Processes*, 50:179–211.

[2] Arbuckle Jr., J. G., Morton, L. W., and Hobbs, J. (2015). Understanding farmer perspectives on climate change adaptation and mitigation: The roles of trust in sources of climate information, climate change beliefs, and perceived risk. *Environment and Behavior*, 47(2):205–234.

[3] Dang, H., Li, E., and Bruwer, J. (2012). Understanding climate change adaptive behaviour of farmers: An integrated conceptual framework. *The International Journal of Climate Change: Impacts and Responses*, 3(2):255–272.

[4] Grothmann, T. and Patt, A. (2005). Adaptive capacity and human cognition: The process of individual adaptation to climate change. *Global Environmental Change*, (15):199–213.

[5] Lopes, L. L. and Olden, G. C. (1999). The role of aspiration level in risky choice: A comparison of cumulative prospect theory and sp/a theory. *Journal of Mathematical Psychology*, 43:286–313.

[6] Lucas, R. E. and Stark, O. (1985). Motivations to remit: Evidence from Botswana. *Journal of Political Economy*, (93).

[7] Luitel, D. R., Jha, P. K., Siwakoti, M., Shrestha, M. L., and Munniappan, R. (2020). Climatic trends in different bioclimatic zones in the Chitwan Annapurna Landscape, Nepal. *Climate*, 136(8).

[8] Massey, D. S., Arango, J., Hugo, G., Kouaouci, A., Pellegrino, A., and Taylor, J. E. (1993). Theories of international migration: A review and appraisal. *Population and Development Review*, 19(3):431–466.

[9] Rogers, R. and Prentice-Dunn, S. (1997). *Handbook of Health Behavior Research 1: Personal and Social Determinants*, chapter Protection Motivation Theory, pages 113–132. Plenum Press.

[10] Stark, O. and Bloom, D. E. (1985). The New Economics of Labor Migration. *The American Economic Review*, (75).

[11] Thapa, R. and Dhakal, S. C. (2024). Climate change perception and adaptation strategies of rice seed groweres in Chitwan District, Nepal. *Farming System*, 2.

[12] Tversky, A. and Kahneman, D. (1991). Loss aversion in riskless choice: A reference-dependent model. *The Quarterly Journal of Economics*, 106(4):1039–1061.
